# Microbiota-derived tryptophan metabolism and AMPK/mTOR pathway mediate antidepressant-like effect of Shugan Hewei Decoction

**DOI:** 10.3389/fphar.2024.1466336

**Published:** 2024-09-16

**Authors:** Yingying Yue, Youlan Ke, Junping Zheng, Zicheng Wang, Hongtao Liu, Songlin Liu

**Affiliations:** ^1^ College of Traditional Chinese Medicine, Hubei University of Chinese Medicine, Wuhan, China; ^2^ Hubei Shizhen Laboratory, Wuhan, China; ^3^ School of Basic Medical Sciences, Hubei University of Chinese Medicine, Wuhan, China

**Keywords:** depression, gut microbiota, tryptophan metabolism, AMPK/mTOR pathway, autophagy, Shugan Hewei Decoction

## Abstract

**Introduction:**

Depression is a common psychological disorder, accompanied by a disturbance of the gut microbiota and its metabolites. Recently, microbiota-derived tryptophan metabolism and AMPK/mTOR pathway were found to be strongly linked to the development of depression. Shugan Hewei Decoction (SHD) is a classical anti-depression traditional Chinese medicine formula. Although, we have shown that SHD exerted antidepressant effects via cecal microbiota and cecum NLRP3 inflammasome, the specific mechanism of SHD on metabolism driven by gut microbiota is unknown. In this study, we focus on the tryptophan metabolism and AMPK/mTOR pathway to elucidate the multifaceted mechanisms of SHD.

**Methods:**

Male rats were established to the chronic unpredictable stress (CUS)/social isolation for 6 weeks, and SHD-L (7.34 g/kg/d), SHD-H (14.68 g/kg/d), Fructooligosaccharide (FOS) (3.15 g/kg/d) were given by intragastric administration once daily during the last 2 weeks. Behavioral experiments were carried out to evaluate the model. The colonic content was taken out for shotgun metagenomic sequencing combined with the untargeted metabolomics, the targeted tryptophan metabolomics. ELISA was used to detect the levels of zonula occludens 1 (ZO-1), Occludin in colon, as well as lipopolysaccharide (LPS), diamine oxidase (DAO), D-lactate (DLA) in serum. The expressions of mRNA and proteins of adenosine monophosphate-activated protein kinase (AMPK)/mammalian target of rapamycin (mTOR) pathway of autophagy were examined using RT-qPCR and Western blot in colon.

**Results:**

SHD modulated gut microbiota function and biological pathways, which were related to tryptophan metabolism. In addition, SHD could regulate microbiota-derived tryptophan production (such as reduction of 3-HK, 3-HAA etc., increment of ILA, IAA etc.), which metabolites belong to kynurenine (KYN) and indole derivatives. Further, SHD reduced intestinal permeability and enhanced the intestinal barrier function. Moreover, SHD could upregulate the levels of AMPK, microtubule associated protein light chain 3 (LC3), autophagy related protein 5 (ATG5) and Beclin1, downregulate the levels of mTOR, p62, promoted autophagy in colon. Spearman’s analysis illustrated the close correlation between tryptophan metabolites and intestinal barrier, AMPK/mTOR pathway.

**Conclusion:**

SHD may exert antidepressant-like effects by regulating microbiota-derived tryptophan metabolism, and triggering the AMPK/mTOR pathway of autophagy, enhancing the intestinal barrier function.

## 1 Introduction

Depression is a common psychological disorder, with a global prevalence of 4.4%, which is the major cause of suicide (Marwaha et al., 2023). With depressed mood and anhedonia as the core symptoms, depression is often accompanied by gastrointestinal dysfunction and other physical symptoms (Malhi and Mann, 2018). The pathogenesis of depression is complex, mainly involving the hypotheses of monoamine, hypothalamic–pituitary–adrenal axis changes, inflammation, neuroplasticity and neurogenesis, and gut microbiome (Cruz-Pereira et al., 2020). Currently, long-term use of first-line antidepressants is prone to significant side effects and further aggravates gastrointestinal symptoms (Berwian et al., 2017). Novel and alternative treatments for patients with depression are urgently needed.

The gut microbiota disruption has been found in depression patients and rodent models (Li et al., 2023; Simpson et al., 2021). Increasing clinical researches have emphasized that compositional and functional dysbiosis in gut microbiota, and their metabolites are involved in the occurrence and progression of depression through the gut-brain axis (Liu D. et al., 2023). The intestinal barrier is in close contact with gut microbiota, its metabolites, and mounting evidence suggests that the intestinal barrier is compromised in depression (Pellegrini et al., 2023; Trzeciak and Herbet, 2021). The intestinal barrier function is influenced by gut microbiota-based metabolites (eg. tryptophan, short-chain fatty acids, bile acids) (Liu L. et al., 2023; Gasaly et al., 2021; Liu D. et al., 2023). It has been reported that the gut microbiota participated in modulating tryptophan metabolism, with subsequent effects on intestinal barrier function and depression (Gao et al., 2020). Therefore, altering the microbiota-derived tryptophan metabolites has become an innovative and potential strategy for the treatment of depression.

Additionally, autophagy and its regulatory mechanisms are involved in maintaining the intestinal epithelium and supporting intestinal barrier function through tight junction (TJs) proteins (Foerster et al., 2022). It should be noted that the adenosine monophosphate-activated protein kinase (AMPK)/mammalian target of rapamycin (mTOR) pathway is a vital regulatory target in autophagy (Guo et al., 2022). Existing studies have shown that the microbial tryptophan metabolites, such as kynurenine (KYN), kynurenic acid (KYNA), and indole-3-lactate (ILA) can regulate AMPK/mTOR-mediated autophagy to influence intestinal barrier (Fan et al., 2020; Gao et al., 2022; Ling et al., 2023). So far, few studies have explored the link between tryptophan metabolism in gut microbiota and AMPK/mTOR pathway of autophagy during the pathology of depression.

Chinese herbal compound has been successfully used to relieve depression by affecting various targets (Zhou et al., 2018). Shugan Hewei Decoction (SHD) is a traditional Chinese medicine formulation, which has remarkable clinical efficacy in treating depression (Li et al., 2023; Mou et al., 2019). Although, our previous studies found that SHD could alleviate depression-like behaviors via modulating the composition of cecal microbiota and restraining the NOD-like receptor protein 3 inflammasome (Yue et al., 2021). As a barrier tissue, the colon is more reflective of microbial metabolites and disease characteristics than the cecum (James et al., 2020). However, the mechanism of how SHD exerts its antidepressant effects by adjusting the microbial tryptophan metabolism to maintain the intestinal barrier remains unclear. Here, using the rat model with chronic unpredictable stress (CUS) and social isolation, we reveal the regulatory network of SHD on gut microbiota-derived tryptophan metabolites, suggesting SHD could exert antidepressant-like effect by modulating microbial tryptophan metabolism and AMPK/mTOR pathway.

## 2 Materials and methods

### 2.1 Reagents and materials

E.Z.N.A.^®^ Soil DNA kit was produced by Omega Bio-Tek, Inc. (Norcross, GA, United States). ELISA kits for lipopolysaccharide (LPS), zonula occludens 1 (ZO-1) and Occludin were manufactured from Cusabio Inc. (Wuhan, China, #CSB-E14247r, #CSB-E17287r, #CSB-E17291r). ELISA kits for diamine oxidase (DAO) and d-lactate (D-LA) were manufactured by Elabscience Biotechnology Co. (Wuhan, China, #E-BC-K524-M) and Quanzhou Ruixin Biological Technology Co. (Quanzhou, China, #RXJ303122R), respectively. Antibodies for including p-AMPK(#ab133448), AMPK(#ab32047), microtubule associated protein light chain 3 (LC3) (#ab192890), GAPDH (#ab8245) were obtained from Abcam (Cambridge, United Kingdom). Beclin 1 and p-mTOR, mTOR were obtained from Proteintech (Wuhan, China, #11306-1-AP, #67778-1-Ig, #66888-1-Ig). Primary antibody to ZO-1 was purchased from Bioss (Beijing, China, #bs-34023R), Occludin was purchased from Santa Cruze (California, United States, #sc-133256). Other antibodies including autophagy related protein 5 (ATG5) and p62 were purchased from Affinity Biosciences (Changzhou, China, #DF7579, #AF5384). Dry fructooligosaccharide (FOS) (purity ≥ 95.0%, No. 2010192134) was obtained from Quantum Hi-Tech Biological Co. Ltd. (Guangzhou, China). Methanol (#A452-4, purity ≥ 99.9%) and acetonitrile (#A998-4, purity ≥ 99.95%) were purchased from Affinity Biosciences Thermo Fisher Scientific Inc. (Massachusetts, United States).

### 2.2 Drug preparation

SHD comprises ten herbs, including Bupleuri Radix (Chaihu, *Bupleurum chinense* DC., 10 g, root and rhizome, Lot: 20200401), Paeoniae Radix Alba (Baishao, *Paeonia lactiflora* Pall., rhizome, 10 g, Lot: 202009010), Aurantii Fructus Immaturus (Zhishi, *Citrus × aurantium* L., 10 g, fructus, Lot: 2020006102), Curcumae Radix (Yujin, *Curcuma aromatica* Salisb., 10 g, root, Lot: 202009015), Amomi Fructus [Sharen, *Wurfbainia villosa* (Lour.) Skornick. and A.D.Poulsen, 10 g, fructus, Lot: 2020004105], Atractylodis Macrocephalae Rhizoma (Baizhu, *Atractylodes macrocephala* Koidz., rhizome, 15 g, Lot: 200702), Aucklandiae Radix (Muxiang, *Aucklandia costus* Falc., root, 10 g, Lot: 202008014), Coptidis Rhizoma (Huanglian, *Coptis chinensis* Franch., rhizome, 6 g, Lot: 2020912102), Euodiae Fructus [Wuzhuyu, *Tetradium ruticarpum* (A.Juss.) T.G.Hartley, fructus, 6 g, Lot: 2020912104], Glycyrrhizae Radix et Rhizoma (Gancao, *Glycyrrhiza uralensis* Fisch. ex DC., root and rhizome, 6 g, Lot: 202009004) were obtained from Tianji Pharmaceutical Co., Ltd. (Hubei, China). All of the herbal materials were identified by Prof. Zeng Xiangfa from the School of Basic Medical Sciences, Hubei University of Chinese Medicine, based on the Chinese Pharmacopoeia 2020 edition (National Pharmacopoeia Commission, 2020). All specimens were stored in the Key Laboratory of Chinese Medicine Resources and Chinese Medicine Compounds of Hubei University of Chinese Medicine, China.

Our previous study (Yue et al., 2021) described the preparation details of SHD and investigated the quality control of SHD by HPLC-Q-TOF-MS/MS with the identical formulation. The HPLC-Q-TOF-MS/MS analysis of ingredients from the SHD sample were showed in Supplementary Figure S1 and Supplementary Table S1. The FOS was administered intragastrically in the dosage of 3.15 g/kg/day (Chi et al., 2020). The dosage of SHD-L and SHD-H were 7.34 g/kg/d and 14.68 g/kg/d respectively. It should be note that the dosage of SHD-L was the clinical equivalent. The dosing calculation in this study was according with the screened dominant dose in our previous studies (Yue et al., 2021) and the FDA guidelines for the converting of animal dose to human equivalent dose. The drug preparation details were described in Supplementary Materials.

### 2.3 Animal care and use

All animal experiments were approved by the Ethics Committee of the Hubei University of Chinese Medicine (HUCMS 00285145). This study involving animals was conducted conforming to the European Community guidelines. Eighty-six male Sprague–Dawley SPF rats (190–200 g) were purchased from Hubei Provincial Center for Disease Control and Prevention (Wuhan, China) and housed in the Hubei University of Chinese Medicine’s SPF Animal Lab. Under the controlled experimental environment (temperature: 24°C ± 1°C, humidity: 60% ± 5%), all rats were acclimatized with 12 h of light/darkness each day with an unrestricted supply of food and water.

After habituation of 7 days, the rats with large weight differences were eliminated and 81 rats with a similar weight were screened. Rats were randomly divided into two groups: the control group (n = 15) and the CUS model group (n = 66). The control group was housed in one cage for every 5 rats, but the CUS model group was housed alone for social isolation. The model group was exposed to CUS for 6 weeks, as reported in the previous paper (Supplementary Table S2) (Willner et al., 1987). At the end of 4 weeks, we removed CUS-resistant (10 rats) according to the sucrose preference test (SPT), open field test (OFT), forced swimming test (FST). Then divide the remaining 56 rats into four groups randomly: the CUS group (n = 14), the CUS + SHD-L group (n = 14), the CUS + SHD-H group (n = 14), and the CUS + FOS group (n = 14). In terms of 5–6 weeks, modeling and drug administration were carried out simultaneously. Both CUS + SHD-L, CUS + SHD-H and CUS + FOS were given by intragastric administration once daily, and the dose were selected derived from the effects of our preliminary research for pharmacodynamic reasons and multi-dose study (Yue et al., 2021). The control rats had access to food and water, with no stimulation. The other groups were subjected to CUS stimulation, and the SHD-L, SHD-H and FOS groups received the corresponding interventions.

### 2.4 Behavioral testing

SPT, FST and OFT were performed as previous described to assess the depression-like behaviors (Ye et al., 2023). The details of behavioral testing were described in Supplementary Materials.

### 2.5 Tissue and serum samples collection

After anesthesia (pentobarbital sodium, 50 mg/kg), all rats were sacrificed. Then the blood, colonic tissues, and colonic content were collected under sterile environmental conditions. Serum was obtained from the blood samples with centrifugation (3,000 rpm, 4°C, 10 min), and was stored at −20°C before use. Immediately, all samples were stored at −80°C. The rats were performed for aortic perfusion and fixation by 4% paraformaldehyde. Then colon was separated in 1 cm length, fixed with paraformaldehyde (4°C, 4–6 h).

### 2.6 Shotgun metagenomic sequencing

In brief, DNA extraction from colonic content by the E. Z.N.A.^®^ Soil DNA kit (Omega Bio-tek, Norcross, GA, U.S.) in accordance with manufacturer’s specifications. The concentration and purity of extracted DNA were detected using TBS-380 and NanoDrop2000. DNA was disconnected to a size of 400 bp averagely with Covaris M220 (Gene Company Limited, China) to establish the paired-end library, which was quality tested and prepared by NEXTFLEX Rapid DNA-Seq (Bioo Scientific, Austin, TX, United States). Attached the adapters, which were comprising the complete sequencing primer hybridization sites, to the blunt-end of fragments. The paired-end library was followed by sequencing on the Illumina NovaSeq 6,000 (Illumina Inc., San Diego, CA, United States), and then paired-end sequencing was performed by NovaSeq Reagent kits in accordance with the instructions (www.illumina.com) at Majorbio Bio-Pharm Technology Co., Ltd. (Shanghai, China). Store and save the sequence data in NCBI Short Read Archive database (Accession Number: SRP466650). Subsequent steps and methods, such as gene prediction, taxonomy, and functional annotation were described in Supplementary Materials.

### 2.7 Metabolomics

#### 2.7.1 Liquid chromatography-mass/mass spectrometry (LC-MS/MS) untargeted metabolomics

Metabolites were extracted from the colonic content samples (50 mg) in 400 µL methanol: water (4:1, v/v) solution and 0.02 mg/mL L-2-chlorophenylalanine using the high-throughput tissue grinder Wonbio-96c (Shanghai Wonbio Biotechnology Co., Ltd.). The mixture was precipitated at −10°C and homogenized using Wonbio-96c (50 Hz, 6 min), followed by ultrasound (40 kHz, 30 min, 5°C). Sample was placed at −20°C for 30 min to precipitate the protein. The mixture was centrifuged (13,000 g, 15 min, 4°C) and the supernatant was transferred to the sample bottle for LC-MS/MS analysis. The instrument platform used for analysis was the UHPLC-Q Exactive HF-X system. The Supplementary Materials described the chromatographic conditions, MS conditions, data preprocessing and annotation, and differential metabolite analysis for LC-MS/MS analysis.

#### 2.7.2 Tryptophan metabolism by LC–MS/MS targeted metabolomics

In brief, weigh 25 mg of samples from colonic content and then add 10 μL internal standard working fluid and 390 μL extraction solution (methanol -water solution, 4:1) sequentially. After that, grind them at −40°C for 6 min (Wonbio-96E, Shanghai Wonbio Biotechnology Co., Ltd.), sonicate at 5°C for 30 min (40 kHz) and stewing at −20°C for 30 min. Following that centrifugate them at 13,000 rcf and 4°C for 15 min, and take the supernatant 280 μL to be dried by nitrogen stream. Then add 0.1% formic acid in acetonitrile 70 μL, centrifugate secondly, take the supernatant for the LC-MS/MS analysis. QTRAP 6,500+ system was used to analysis as the instrument platform. The Supplementary Materials described the chromatographic conditions, MS conditions, data acquisition and processing for LC-MS/MS analysis.

### 2.8 ELISA

The levels of LPS, D-LA and DAO in serum and the contents of ZO-1, Occludin in colonic tissue were assessed. The colonic tissue was washed with PBS, homogenized, and centrifuged (5,000 rpm, 10 min). The resulting supernatant and serum were used as the substrate, and all the above indicators were tested by commercial ELISA kits according to the manufacturer’s instructions.

### 2.9 Immunohistochemistry

The colonic tissue slides were stained with antibodies against ZO-1 and Occludin. Primary antibodies to ZO-1 and Occludin were utilized in the dilution of 1:100. Slides were deparaffinized with xylene and rehydrated through graded series of ethanol, then subjected to antigen retrieval via microwaving in 2 × 8 min with citrated acid buffer. Subsequently, the immunohistochemical staining kit, primary, and secondary antibodies were tested following the instructions provided by the manufacturer state. Thereafter, the incubation with DAB was conducted. Finally, the sections were counterstained in Siwega’s hematoxylin for 3 min before being examined via a fluorescence microscope. A positive index of ZO-1, Occludin in the colonic tissue were observed by Leica AF6000 and quantified under ImageJ software in randomly selected areas.

### 2.10 Quantitative real-time PCR

The expressions of AMPK, mTOR, LC3, ATG5, Beclin1 and p62 genes were determined. Total RNA was extracted from the colon by trizol reagents (Solarbio, Beijing, China, #R1100) referring to vendor’s instructions, then concentration and purity were determined. The relative expressions of genes were calculated by 2^−ΔΔ^Ct method, and GAPDH mRNA level was used to standardize the target mRNA. Primer sequences were shown in Supplementary Table S3.

### 2.11 Western blot

Protease inhibitor-containing lysis buffer made from RIPA was used for separating colonic tissue protein. BCA kit was used to measure the concentrations of proteins. 50 μg protein samples were separated using 10% SDS-PAGE, and transferred to polyvinylidene difluoride membranes. After the membranes being blocked with 5% skim milk (1 h, 37°C), the proteins were subjected to incubation with the succeeding primary antibodies overnight (4°C). The membranes were incubated with secondary antibodies for 1 h at room temperature. ECL chemiluminescent solution and gel imaging system were used to visualize the blot bands. As below there were the details of primary antibodies: p-AMPK (1:5,000), AMPK (1:2,000), p-mTOR (1:5,000), mTOR (1:20,000), LC3 (1:2,000), ATG5 (1:2,000), Beclin1 (1:5,000), p62 (1:1,000) and GAPDH (1:10,000). The protein levels were quantified by ImageJ software processing system.

### 2.12 Statistical analysis

GraphPad Prism 9.0 software (GraphPad Software, Inc., San Diego, CA, United States) was used to analyze the quantitative data, and the date were described with mean ± SEM. Comparisons of four groups were processed with students’ t-test for normally distributed data, and Wilcoxon rank sum-test and Kruskal–Wallis H test for non-normally distributed data. The correlation analysis between the metabolites and gut microbiota, autophagy, mucosal barrier related indicators was analyzed by Spearman. *p* < 0.05 was indicated a significant difference, *p* < 0.01 was indicated a greatly significant difference. Both were considered statistically significant.

## 3 Results

### 3.1 SHD ameliorates depression-like behaviors

**FIGURE 1:**
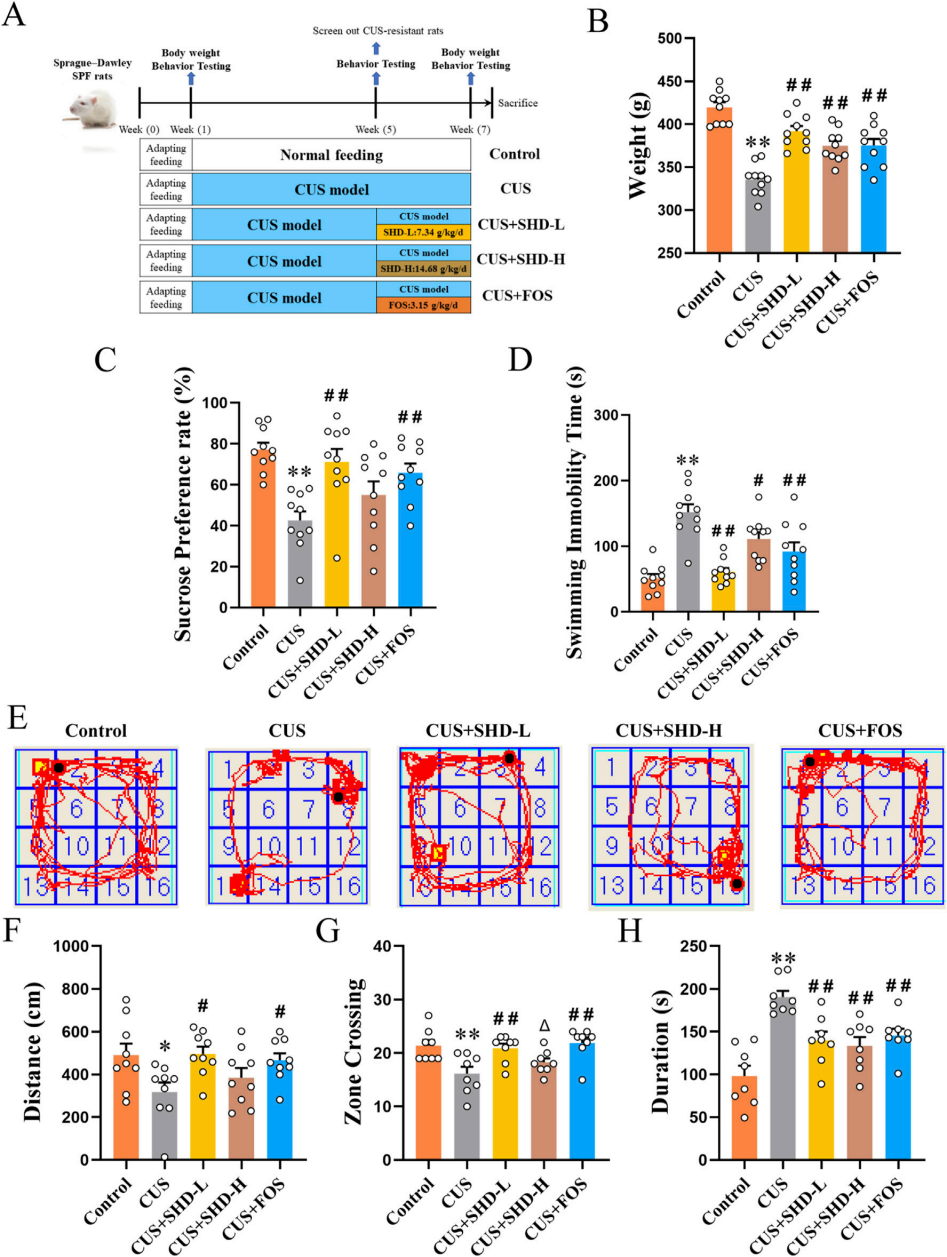
SHD ameliorates depression-like behaviors in CUS rats. **(A)** Experimental arrangement and animal grouping. **(B)** The weight of rats at the 6th week (42 days) in each group (n = 10). **(C)** Sucrose preference rate from SPT (n = 10). **(D)** Immobility time in FST (n = 10). **(E)** Trajectory of rat within 5 min in OFT. **(F)** Total movement distance in OFT (n = 9). **(G)** Number of crossing central areas in OFT (n = 8). **(H)** Stationary time in OFT (n = 8). Data are presented with mean ± SEM. *p < 0.05, **p < 0.01 compared to control group. ^#^ *p* < 0.05, ^##^ *p* < 0.01 compared to CUS group. ^Δ^ *p* < 0.05, ^ΔΔ^ *p* < 0.01 compared to CUS + FOS group.

Experimental arrangement and animal grouping were conducted according to Figure 1A. These findings suggested that the rats had weight loss in the CUS group compared with the control group (*p* < 0.01). The SHD-L, SHD-H, FOS intervention partially restored the body weight in CUS group (*p* < 0.01, respectively, Figure 1B; Supplementary Table S4). SPT, FST and OFT were tested to determine the antidepression effects of SHD in CUS-induced rats. The sucrose preference was decreased in the CUS (*p* < 0.01). In contrast, the SHD-L, FOS increased sucrose preference compared to the CUS group (*p* < 0.01, respectively, Figure 1C; Supplementary Table S4), whereas SHD-H was not statistically significant (*p* > 0.05). As shown in FST (Figure 1D), the CUS group showed greater swimming immobility duration (*p* < 0.01), whereas, the administration of SHD-L, SHD-H and FOS significantly shortened the immobility duration (*p* < 0.01 or *p* < 0.05, Supplementary Table S4).

Following CUS stimulation, there was a notable trend of decreased total movement distance (*p* < 0.05), number of crossing central areas (*p* < 0.01), and rised stationary time (*p* < 0.01). In contrast, the treatment with SHD-L or FOS could significantly reverse the decrease of total movement distance (*p* < 0.05, respectively), the number of crossing central areas (*p* < 0.01, respectively) and the increase of stationary duration (*p* < 0.01, respectively) compared to CUS. However, the SHD-H only could reverse the increase of stationary duration (*p* < 0.01). As demonstrated by the results, the SHD-L and FOS can achieve good efficacy in depression-like behaviors induced by CUS (Figures 1E–H; Supplementary Table S5), and the SHD-L was clinically equivalent dose according to pharmacodynamic research and clinical use. Thus the SHD-L (7.34 g/kg/d) was chosen for the subsequent experimental study of SHD.

### 3.2 SHD modulates gut microbiota function and biological pathways

**FIGURE 2:**
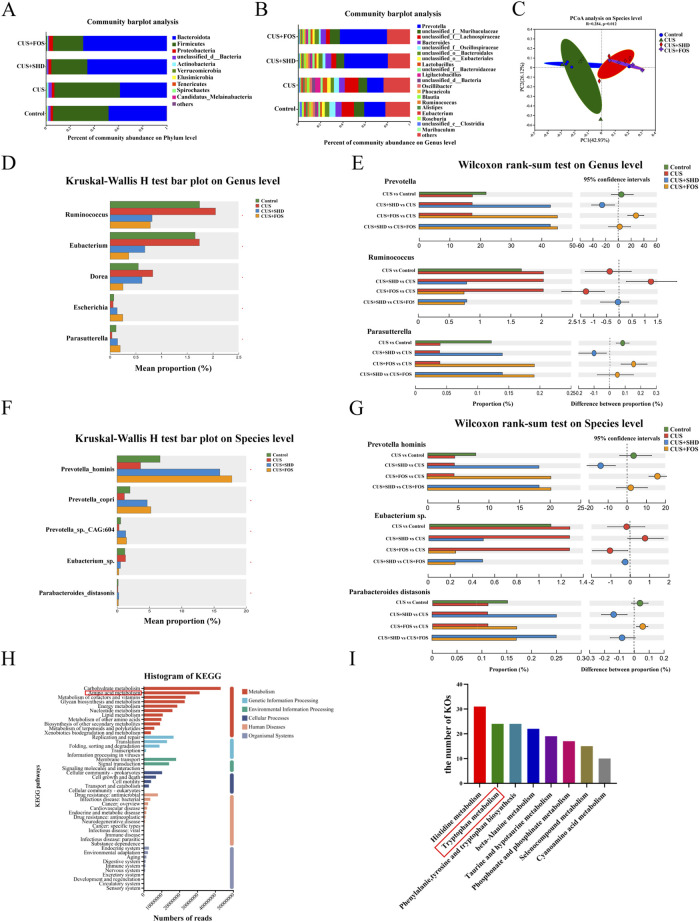
SHD modulates gut microbiota, microbiota function and biological pathways in CUS rats. **(A)** Relative microbiota abundance at phylum level. **(B)** Relative microbiota abundance at genus level. **(C)** PCoA analysis on species level. **(D)** Comparison of the relative microbiota abundance from the control, CUS, CUS + SHD and CUS + FOS groups at genus level. **(E)** Bar plot at the genus level of Prevotella, Ruminococcus and Parasutterella. **(F)** Comparison of the relative microbiota abundance from the control, CUS, CUS + SHD and CUS + FOS groups on species level. **(G)** Bar plot on the species level of Prevotella hominis, Eubacterium sp. and Parabacteroides distasonis. **(H)** Annotations of KEGG functional category. **(I)** The number of KOs related to amino acids. The data are expressed as the mean ± SD, n = 4. *Represents statistical significance, *p < 0.05.

Although our previous studies used 16S rRNA gene sequencing to detect the composition and diversity of cecal microbiota, the specific microbial function, gene composition, related metabolic pathway driven by gut microbiota is unknown. So in this study, we detected the colonic contents focusing on the function and biological pathways of biomarker in microbiota by shotgun metagenomic sequencing to explore the potential mechanism. The treatment with SHD and FOS showed an increasing trend in Bacteroidota, but a downward trend in the abundance of Firmicutes, Actinobacteria compared to the CUS model group (Figure 2A). Dysbiosis in CUS model rats was characterized by a decrease of *Prevotella*, *Parabacteroides*, and an expansion of *Lactobacillus*, *Ligilactobacillus*, *Blautia* and *Ruminococcus*. In contrast, the abundance of *Prevotella*, *Parabacteroides* were substantially upregulated and *Lactobacillus*, *Ligilactobacillus*, *Blautia* and *Ruminococcus* were significantly downregulated, after the treatment with SHD or FOS (Figure 2B). The PCoA analysis on species level (*R* = 0.284, *p* = 0.012) showed that the difference in community composition among four grouped samples is more remarkable than the difference within groups (Figure 2C). Moreover, significantly differential phylum relative microbiota abundance was demonstrated in the different groups (Supplementary Figure S2). At the genus level (Figures 2D, E), the results displayed that the treatment of SHD or FOS could reverse the alteration of microbiota in the CUS model, including increasing the abundance of *Prevotella*, *Escherichia*, *Parasutterella*, and reducing *Ruminococcus*, *Eubacterium*, *Dorea*. At the species level (Figures 2F, G), with the treatment of SHD or FOS, not only the relative abundance of *Eubacterium sp.* was markedly reduced, but also the *Prevotella hominis*, *Prevotella copri*, *Prevotella sp. CAG:604*, *Parabacteroides distasonis* were significantly increased.

According to the KEGG database, the data obtained from metagenomic analysis were annotated to observe the influence of SHD in gut microbiota function and biological pathways. The results demonstrated that the top five functional categories of abundance were closely related to metabolism, including carbohydrate, amino acid, cofactors and vitamins, glycan biosynthesis, and energy metabolism (Figure 2H). Among, the results of KEGG orthologues (KOs) related to amino acids indicated that the top three were histidine metabolism, tryptophan metabolism, phenylalanie, tyrosine and tryptophan biosynthesis (Figure 2I).

### 3.3 SHD regulates tryptophan metabolism in colonic contents

**FIGURE 3:**
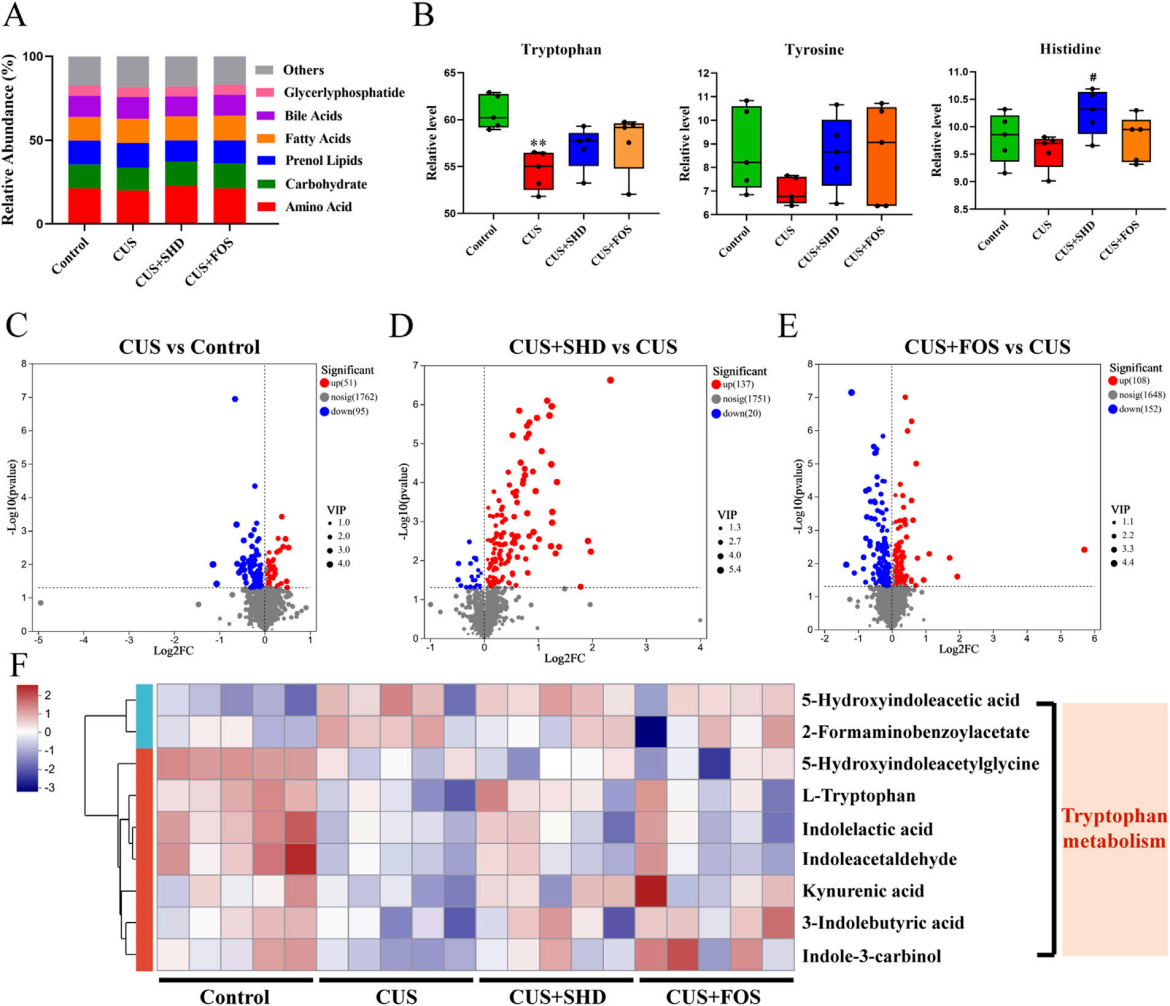
SHD regulates metabolism in colonic contents of CUS rats by untargeted metabolomics analysis. **(A)** Relative abundance of metabolite category in each group. **(B)** Relative abundance of metabolite of tryptophan, tyrosine and histidine in each group. Data are presented with mean ± SEM. *p < 0.05, **p < 0.01 compared to control group. ^#^ *p* < 0.05, ^##^ *p* < 0.01 compared to CUS group. ^Δ^ *p* < 0.05, ^ΔΔ^ *p* < 0.01 compared to CUS + FOS group. **(C)** Volcano plot of the CUS group vs. control group. **(D)** Volcano plot of the SHD group vs. CUS group. **(E)** Volcano plot of the FOS group vs. CUS group. *p* < 0.05 was indicated a significant difference. FC (Fold Change) = 1. **(F)** The heatmap of differential metabolites related to tryptophan in each group. Blue and red mean the negative or positive correlation, respectively.

Composition and abundance changes in gut microbiota will result in alterations of gut microbial metabolites and functions (Zhang et al., 2023). To further identify the influence of SHD on gut microbial metabolites, non-targeted metabolomics was used to analyze the colonic contents. First, we determined the colonic contents metabolites of the four groups using OPLS-DA and substitution tests. Our study indicated that significant differences were among the four groups (Supplementary Figure S3). Figure 3A and Supplementary Table S6 indicated that the metabolisms of amino acids and carbohydrate was downregulated and the metabolisms of bile acid, prenol lipids, and fatty acids were upregulated in the CUS group while the treatment of SHD or FOS could reverse the results. Then we found that the top three metabolites of amino acids in terms of relative level were tryptophan, tyrosine and histidine (Figure 3B; Supplementary Table S7). All of the above metabolisms of amino acid were downregulated in the CUS group and the treatment of SHD or FOS could reverse the results.

To reveal the differential metabolites between the groups, a combination of VIP>1 and *p* < 0.05 was screened in the OPLS-DA. This result demonstrated that compared to the control group, there were 146 metabolites altered (including 51 upregulated and 95 downregulated) of the model group (Figure 3C). 137 of the altered metabolites were upregulated, whereas 20 of the altered metabolites were downregulated in the SHD group compared with the control group (Figure 3D). The FOS upregulated 108 metabolites and downregulated 152 metabolites compared with the control group (Figure 3E). Next, we analyzed the metabolic pathways of the identified differential metabolites. A heat map showed that most part of the tryptophan metabolites were reduced in the CUS model group, while SHD increased the content of the above tryptophan metabolites (Figure 3F). The observation demonstrated that SHD could upregulate the relative level of tryptophan metabolites in the colonic contents.

**FIGURE 4:**
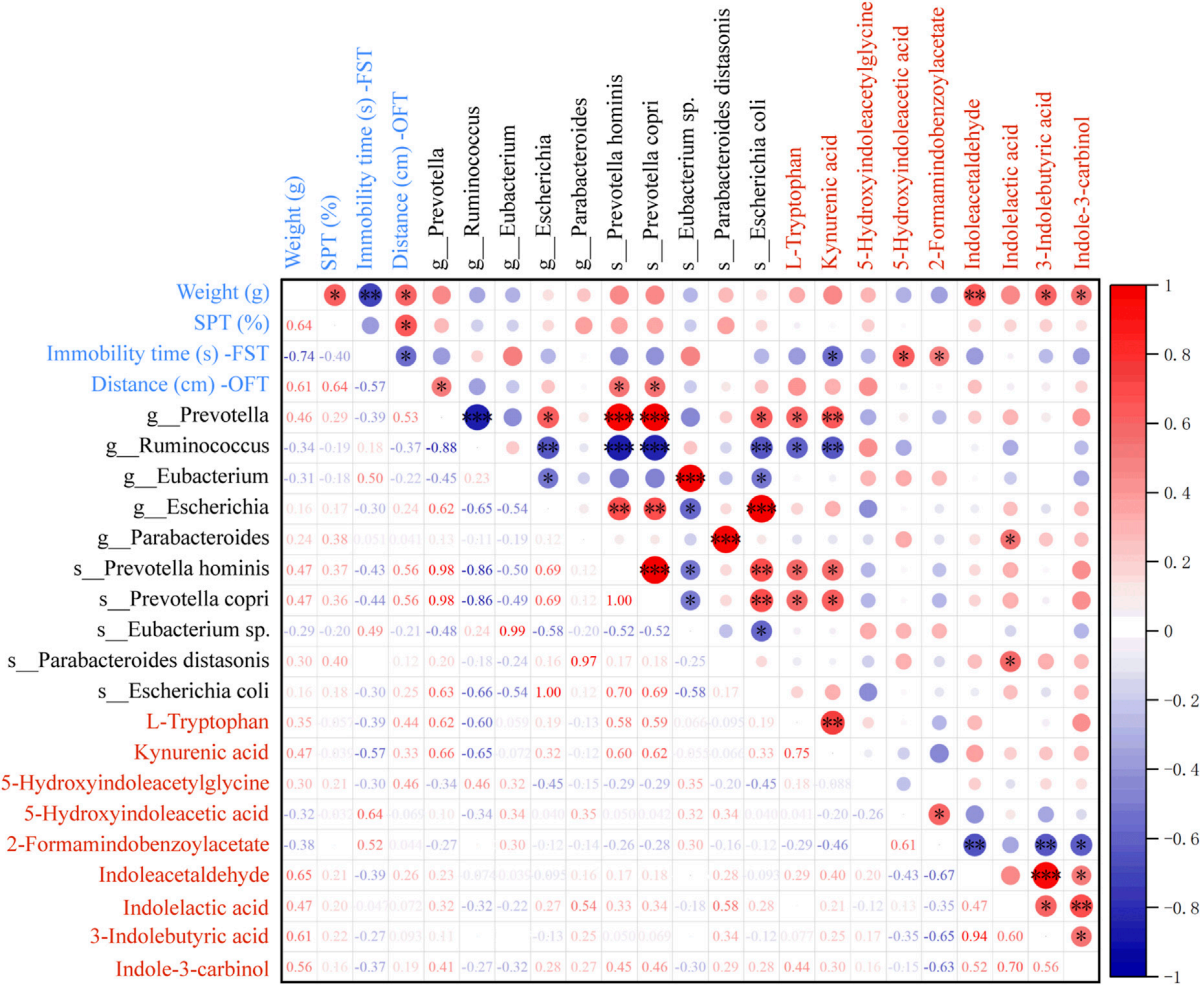
Spearman analysis for the correlation between gut microbiota, depression-like behaviors, and differential metabolites related to tryptophan. R value, the closer this value is to 1, the more positive the correlation, and the closer it is to −1, the more negative. Blue and red mean the negative or positive correlation, respectively. *p < 0.05, **p < 0.01, ***p < 0.001.

To confirm whether the antidepressant mechanism of SHD was linked to gut microbiota through tryptophan metabolism, we studied the correlations between gut microbiota, depression-like behaviors, and tryptophan metabolites (Figure 4). The findings illustrated that the relative levels of Indoleacetaldehyde, 3-Indolebutyric acid, Indole-3-carbinol were positively related to weight, and the relative level of 5-Hydroxyindoleacetic acid was positively linked to FST immobility time. And the level of KYNA was negatively associated with FST immobility time, and positively with OFT distance. Moreover, the levels of L-tryptophan and KYNA were positively linked to the abundance of *Prevotella*, *Prevotella hominis*, *Prevotella copri*. The level of KYNA was negatively related to the abundance of *Ruminococcus*. And the level of indolelactic acid was positively related to the abundance of *Parabacteroides* and *Parabacteroides distasomis*. These results indicated that SHD may play an antidepressant role by adjusting tryptophan metabolism in colonic contents, and the gut microbiota could participate in tryptophan metabolism.

**FIGURE 5:**
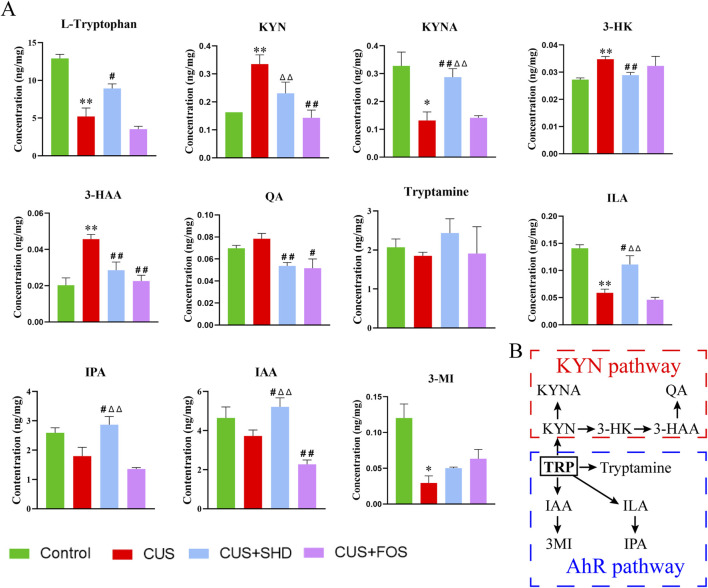
SHD regulates tryptophan metabolism in colonic contents in each group by the targeted metabolomics analysis. **(A)** Quantification of tryptophan and its metabolites in mice colonic content. **(B)** Pathways of tryptophan metabolism. The data are expressed as the mean ± SDM, n = 4. *p < 0.05, **p < 0.01 compared to control group. ^#^ *p* < 0.05, ^##^ *p* < 0.01 compared to CUS group. ^Δ^ *p* < 0.05, ^ΔΔ^ *p* < 0.01 compared to CUS + FOS group. 3-HAA, 3-hyrdoxyanthranilic acid; 3-HK, 3-hydroxy-kynurenine; 3-MI, 3-methylindole; IAA, indole-3-acetic acid; ILA, indole-3-lactic acid; IPA, indole-3-propionic acid; KYN, kynurenine; KYNA, kynurenic acid; QA, quinolinic acid.

Combined with the results of metagenomic sequencing and LC-MS/MS untargeted metabolomics, it is suggested that gut microbiota and its differential metabolites were mainly enriched in tryptophan metabolism. These results implied that tryptophan metabolism may be a key pathway for the pharmacological effect of SHD. So next targeted metabolomics was used to test the level of tryptophan metabolites in colonic contents (Figure 5). The levels of L-tryptophan, 3-methylindole (3-MI), ILA, KYNA in the model group reduced compared to the control group, while the levels of KYN, 3-hydroxy-kynurenine (3-HK), 3-hyrdoxyanthranilic acid (3-HAA) rose (*p* < 0.01 or *p* < 0.05). Meanwhile the levels of tryptamine, indole-3-acetic acid (IAA), indole-3-propionic acid (IPA) downregulated and the level of quinolinic acid (QA) upregulated, although without statistical significance. On the contrary, the treatment of SHD could reverse the changes of the above-mentioned metabolites, including reducing the levels of KYN, 3-HK, 3-HAA, QA and increasing the levels of KYNA in KYN pathway, increasing the levels of tryptamine, IAA, 3-MI, ILA, IPA in AhR pathway. Based on the above, we postulated that SHD could inhibit KYN pathway and activate AhR pathway in tryptophan metabolism by regulating gut microbiota to exert antidepressant effects.

### 3.4 SHD enhances intestinal mucosal barrier function

**FIGURE 6:**
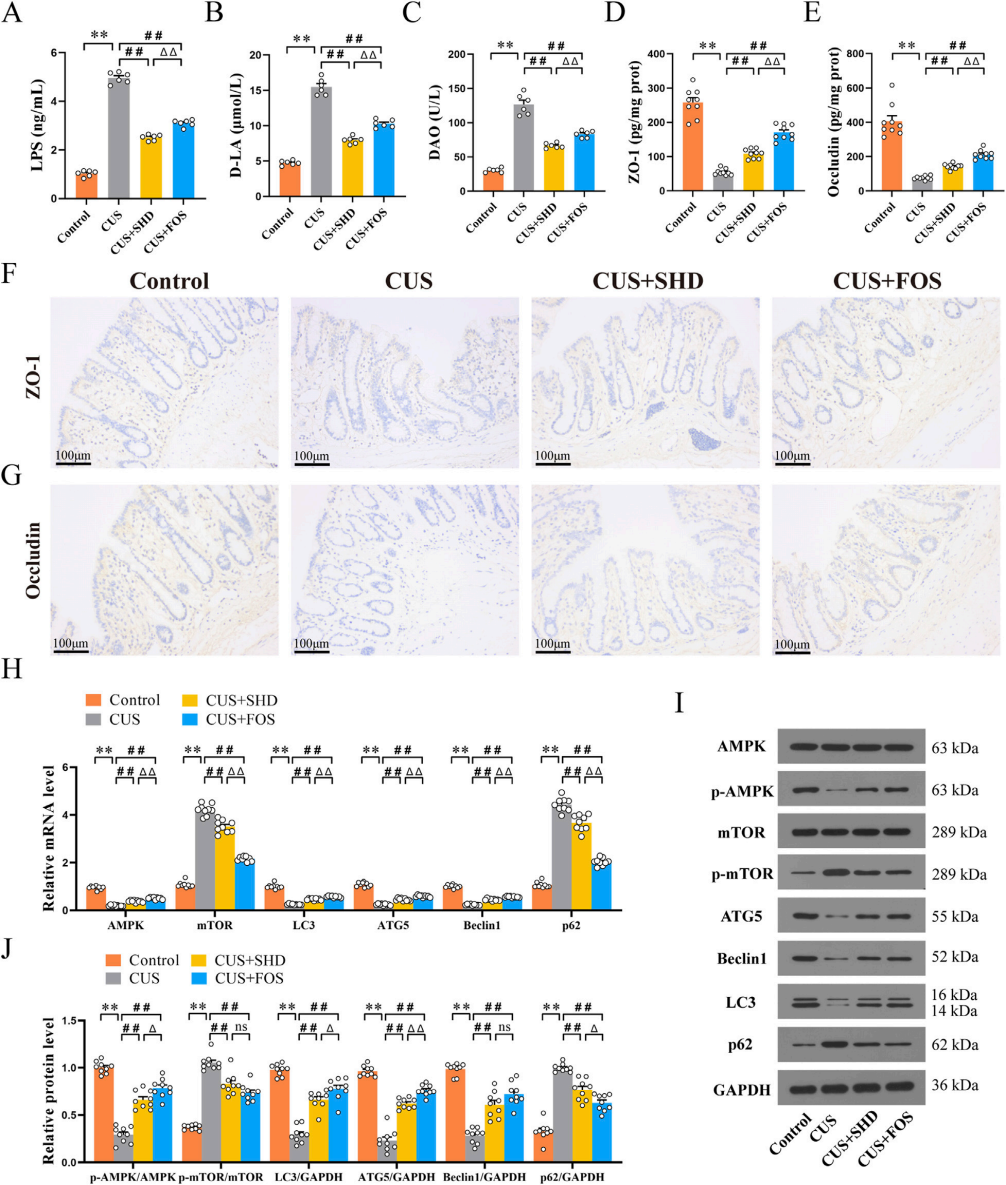
Effect of SHD on colonic barrier function, AMPK/mTOR pathway and autophagy. **(A)** LPS levels of in serum (n = 6). **(B)** D-LA levels in serum (n = 6). **(C)** DAO levels in serum (n = 6). **(D)** The levels of ZO-1 in colonic mucosa (n = 9). **(E)** The levels of Occludin in colonic mucosa (n = 9) **(F)** ZO-1 protein expression in colonic mucosa was quantified by Immunohistochemistry (×200). Scale bar = 100 μm. (n = 4). **(G)** Occludin protein expression in colonic mucosa was quantified by Immunohistochemistry (×200). Scale bar = 100 μm. (n = 4). **(H)** mRNA levels of AMPK, mTOR, LC3, ATG5, Beclin1 and p62 in the colonic tissue (n = 9). **(I)** Quantification of Western blot images (n = 9). **(J)** Protein levels of AMPK, mTOR, LC3, ATG5, Beclin1 and p62. GAPDH as the loading control (n = 9). Data are expressed as the mean ± SEM. Data are presented as mean ± SEM. *p < 0.05, **p < 0.01 compared to control group. ^#^ *p* < 0.05, ^##^ *p* < 0.01 compared to CUS group. ^Δ^ *p* < 0.05, ^ΔΔ^ *p* < 0.01 compared to CUS + FOS group.

For detecting intestinal integrity, higher levels of LPS, D-LA and DAO in serum indicate more severe intestinal damage (Luo et al., 2023). So we examined the contents of LPS, D-LA and DAO in serum to explore the influence of SHD on colonic permeability. The studies (Figures 6A–C; Supplementary Table S8) showed that CUS induced a remarkable enhancement in the contents of LPS, D-LA and DAO (*p* < 0.01) compared with the control group, suggesting that CUS may cause increased colonic permeability induced by mucosal damage. SHD and FOS significantly attenuated the contents of LPS, D-LA and DAO (*p* < 0.01, respectively), and the efficacy of SHD was superior to FOS in reducing colonic permeability (*p* < 0.01). This suggested that SHD could effectively reverse the abnormal increase of colonic permeability.

ELISA and IHC analyses of the colon were performed to determine whether SHD could improve colonic barrier function. TJs can protect the intestinal mucosa from potentially toxic substances to maintain the intestinal barrier. It also acts a key role in maintaining intestinal permeability (Zhao et al. (2021). Therefore, the expressions of tight junction proteins (ZO-1, Occludin) were detected in the colon. Rats exposed to CUS had decreased the level of ZO-1 and Occludin (*p* < 0.01, respectively) (Figures 6D, E; Supplementary Table S9). Similarly, rats in the CUS + SHD and CUS + FOS groups showed a rise in the level of ZO-1, Occludin compared to the CUS group (*p* < 0.01, respectively). The results of ZO-1 and Occludin levels detected using IHC (Figures 6F, G; Supplementary Table S10) were consistent with the results of ELISA. Accordingly, the results suggested that SHD could enhance colonic barrier function via promoting the levels of TJs (ZO-1, Occludin) in CUS model rats.

### 3.5 SHD activates AMPK, inhibits mTOR and promotes autophagy

Autophagy dysfunction is a critical factor in the pathophysiology and progression of intestinal mucosal barrier damage (Foerster et al., 2022) and depression (Gassen and Rein, 2019). Furthermore, dysregulation of expression in mTOR signaling pathways, which modulate autophagy, has been found in victims of depression (Machado-Vieira et al., 2015). Therefore, to evaluate whether SHD influences the AMPK/mTOR autophagy signal pathway, the mRNA levels were detected in the colonic tissues using RT-qPCR. First, we observed the expression of autophagy markers. Figure 6H and Supplementary Table S11 illustrated that the mRNA levels of LC3, ATG5, Beclin1 in the control group were lower than those in the CUS group, but the p62 mRNA level was higher than CUS (*p* < 0.01, respectively). These findings suggest that CUS inhibits the occurrence of autophagy. However, the LC3, ATG5 and Beclin1 mRNA levels were upregulated and the p62 mRNA level were downregulated after the treatment with SHD and FOS (*p* < 0.01, respectively). To further illustrate the molecular mechanism involved in the effects of SHD in CUS model rats, AMPK and its downstream protein mTOR were determined. Compared to the control group, the results demonstrated that the mRNA level of AMPK was downregulated (*p* < 0.01). In contrast, the mRNA level of mTOR was upregulated (*p* < 0.01). And we observed higher AMPK mRNA level and lower mTOR mRNA level after the treatment with SHD and FOS (*p* < 0.01, respectively).

Meanwhile, we evaluated the levels of autophagy pathway associated proteins by Western blot. As shown in Figures 6I, J and Supplementary Table S12, CUS exposure could reduce the protein levels, including p-AMPK, LC3, ATG5, Beclin1, whereas the levels of p-mTOR and p62 were enhanced (*p* < 0.01, respectively). It was worth noting that, after SHD or FOS intervention, the protein levels of p-AMPK, LC3, ATG5, Beclin1 were higher, whereas the protein levels of p-mTOR and p62 were decreased (*p* < 0.01) when compared to that in the CUS. Taken together, SHD and FOS mediate AMPK-mTOR signaling pathway via accelerating the phosphorylation of AMPK to inhibit mTOR activation, which could promote autophagy in the colonic tissues of CUS rats.

### 3.6 Relationship between the tryptophan metabolites and intestinal barrier, AMPK/mTOR pathway

**FIGURE 7:**
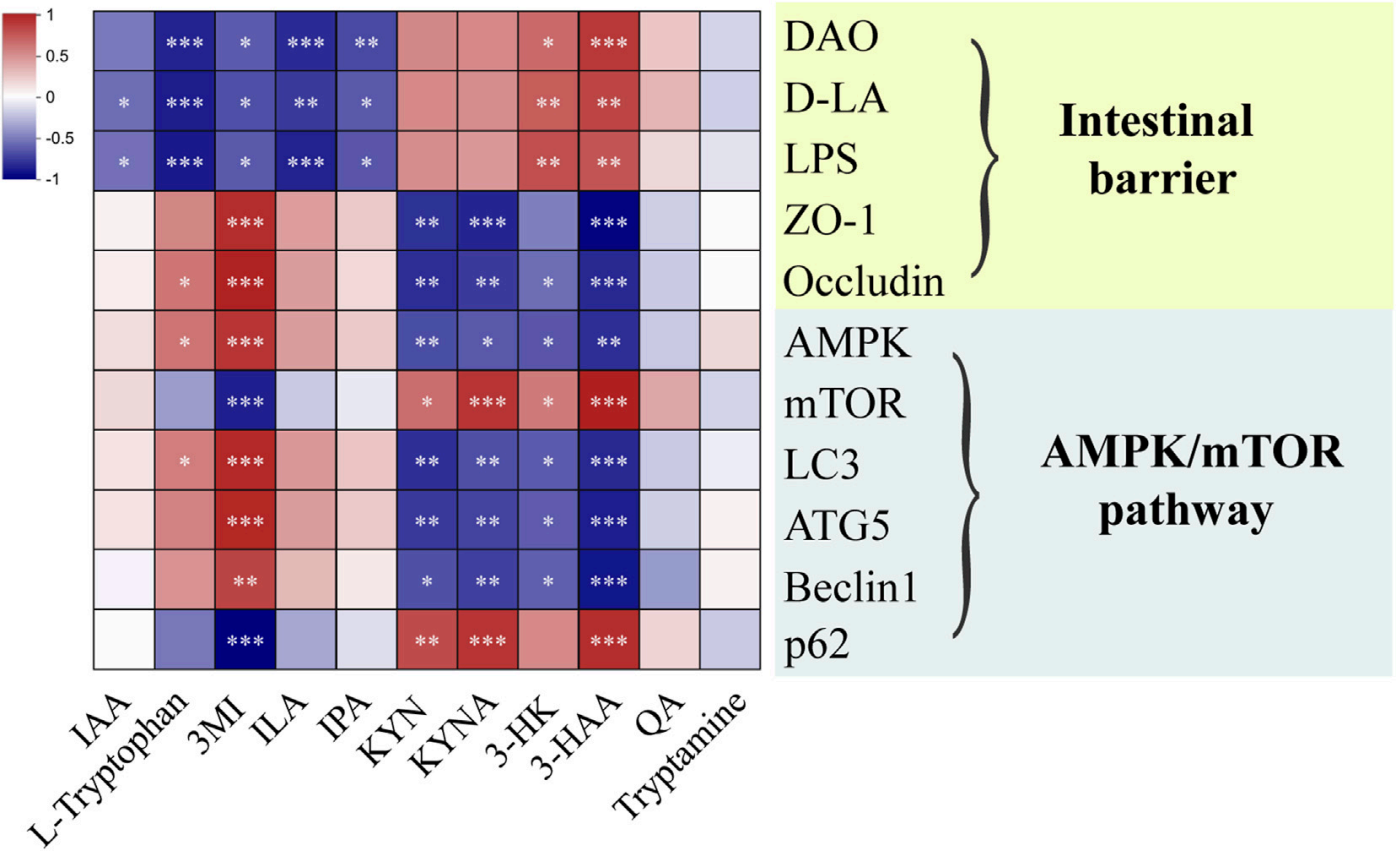
Heat map of the correlation between microbiota-derived tryptophan metabolites and intestinal barrier, AMPK/mTOR pathway of autophagy. Blue and red mean the negative or positive correlation, respectively. *p < 0.05, **p < 0.01, ***p < 0.001.

To confirm whether the mechanism of SHD was linked to the AMPK/mTOR pathway of autophagy through tryptophan metabolism in colonic contents, we analyzed the correlations between tryptophan metabolites and intestinal barrier, the AMPK/mTOR pathway of autophagy by spearman’s correlation test (Figure 7). These results suggested that the expressions of DAO, D-LA and LPS were positively correlated with the levels of 3-HK, 3-HAA and negatively linked to the levels of L-Tryptophan, 3MI, ILA, IPA. The expressions of ZO-1 and Occludin were positively correlated with the level of 3-MI and negatively correlated to the levels of KYN, KYNA, 3-HAA. The mRNA levels of AMPK, LC3, ATG5, Beclin1 were positively associated with the levels of 3-MI and negatively linked to the levels of KYN, KYNA, 3-HK, 3-HAA. In contrast, the mRNA levels of mTOR and p62 were positively related to the levels of KYN, KYNA, 3-HAA and negatively related to the level of 3-MI. These results demonstrated that tryptophan metabolites in colonic contents were closely related to the intestinal barrier function and AMPK/mTOR pathway of autophagy.

## 4 Discussion

Depression is a common mental and psychological disorders and will be the first disease in the global burden of disease by 2030, and often leads to adverse outcomes (McCarron et al., 2021). Growing evidence indicates that depressive disorders could lead to changes in gut microbiota and its metabolites in community of microorganisms throughout the gastrointestinal tract (Nikolova et al., 2021). The gut microbiota regulation of tryptophan metabolism has been shown to be a crucial actor in the intestinal barrier and intestinal mucosa permeability (Wan et al., 2024). In the gut, the three major tryptophan metabolism pathways leading to serotonin (5-hydroxytryptamine), KYN, and indole derivatives are under the direct or indirect control of the microbiota (Agus et al., 2018). In previous studies, we demonstrated that SHD can alleviate depression-like behavior and intestinal mucosal injury by modulating the gut microbiota (Yue et al., 2021), but few studies elaborated light on its therapeutic mechanism on tryptophan metabolism and AMPK/mTOR pathway. In this work, we revealed the correlation between microbiota-derived tryptophan metabolism and AMPK/mTOR pathway to investigate the potential therapeutic effects and the mechanisms of action of SHD in a rat model for CUS. The results indicate that SHD could regulate microbiota-derived tryptophan metabolism and AMPK/mTOR pathway, and enhance the intestinal barrier function to ameliorate depression-like behaviors.

Depression affects heterogeneous cognitive and exploratory behaviors, manifested as anxiety- and depression-like behaviors and other physical symptoms. And the Chinese herbal compounds contain a variety of chemical components, which have the advantage of multi-target and multi-pathway anti-depression. Studies have shown that paeoniflorin in Paeoniae Radix Alba could metabolize into benzoic acid driven by gut microbiota, and thus the metabolites enter the brain through the blood-brain barrier to play an antidepressant effect (Yu et al., 2019). The naringin, hesperidin, neohesperidin from Aurantii Fructus Immaturus, the saikosaponin A from Bupleuri Radix, and the liquiritin from Glycyrrhizae Radix et Rhizoma have been reported to have anti-depressant effects (Wang et al., 2019). In our investigation, HPLC-Q-TOF-MS/MS analysis indicated that seven compounds were distinguished, including paeoniflorin, liquiritin, naringin, hesperidin, neohesperidin, palmatine chloride, saikosaponin A (Supplementary Figure S1). Moreover, SHD reversed the slow weight gain and the behavioral tests also suggested that SHD alleviated the depression-like behavior of rats exposed to CUS (Figure 1). In addition, the dose-response relationship of traditional Chinese medicine is based on clinical equivalent dose and clinical efficacy. In our study, the dosage of SHD-L was the clinical equivalent and the behavioral results showed that the SHD-L has better antidepressant effect than the SHD-H (Figure 1). In current study, it suggested that SHD may play a therapeutic role through the synergistic form of multiple chemical components and targets. Similar antidepressant effects of SHD were also consistent with previous studies (Yue et al., 2021).

Multiple researches indicated that the gut microbiota and microbiota-derived metabolites play a vital part in depression development (Liu L. et al., 2023; Li et al., 2024a). In phyla, Firmicutes, Actinobacteria, and Bacteroidota are the most influenced obviously, and were characterized by an enrichment of the genera *Ruminococcus*, *Desulfovibrio*, *Lactobacillus*, *Blautia* and a depletion of the genera *Prevotella*, *Roseburia*, and *Parabacteroides* of depression (Zheng et al., 2020; Liu D. et al., 2023). *Ruminococcus* within the phylum Firmicutes, have well defined roles in intestinal mucus degradation and immune function, which is able to abolish antidepressants effects on depressive-like behavior (Lukic et al., 2019). Negative associations were observed between DASS-42 scores (depression and anxiety) and Dorea in Firmicutes (Taylor et al., 2020). A recent report revealed that *Prevotella* was depleted in faeces in depression and anxiety co-morbidities in diarrhea-predominant inflammatory bowel disease patients (McGuinness et al., 2022). Additionally, tryptophan-KYN metabolism is thought to be a link between the gut and brain for depression in inflammatory bowel disease (Chen et al., 2021). In agreement with the researches, our current results showed that the treatment with SHD showed an increasing trend in Bacteroidota, but a downward trend in the abundance of Firmicutes, Actinobacteria, particularly by decreasing *Ruminococcus*, *Eubacterium*, *Dorea*, increasing *Escherichia*, *Parasutterella* at the genus level, increasing *Prevotella hominis*, *Prevotella copri*, *Parabacteroides distasonis*, decreasing *Eubaeterium* sp. at the species level (Figures 2A–G). Moreover, the function and biological pathways of differential microbiota were focused on tryptophan metabolism of amino acid metabolism (Figures 2H, I).

On the basis of the gut microbiota composition, about 4%–6% of tryptophan is converted into various intermediates. Interestingly, researches have demonstrated that the disordered gut microbiota can cause tryptophan metabolism disturbance, which damages intestinal mucosal barrier and causes depression (Deng et al., 2021; Ghosh et al., 2021). Tryptophan is an key factor for protecting the integrity and barrier function in the intestinal mucosa (Xie et al., 2024) and metabolites have different kinds of physiological functions, such as repairing intestinal barrier, balancing intestinal microecological, and regulating immune (Li et al., 2022). For example, KYN, a tryptophan metabolite, change leads to a disordered homeostasis of the gut microenvironment, influencing the gut epithelium function and causing intestinal barrier dysfunction to induce depressive symptoms (Li et al., 2022). Tryptophan, as an essential amino acid, could be metabolized into indole, tryptamine, and its derivatives by *Prevotella*, belonging to *Bacteroides* known to produce ILA and IAA (Gasaly et al., 2021; Fernandez-Cantos et al., 2024). Studies have confirmed that *Parabacteroides distasonis* could promote the production of tryptophan metabolite IAA, which could activate the AhR pathway to repair intestinal barrier (Liu L. et al., 2023). *Parasutterella* can influence the aromatic amino acids, which can promote the deamination and chain shortening process in tryptophan metabolism in the colon (Ju et al., 2019). In addition, multiple researches indicated that *Bacteroides thetaiotaomicron*, *Bacteroides eggerthii*, *Bacteroides ovatus*, *Bacteroides fragilis*, *Parabacteroides distasonis* (belonging to Bacteroidetes), and *Eubacterium hallii*, *Clostridium bartlettii* (belonging to Firmicutes) also produced IAA (Russell et al., 2013). In our research, we reveal that after SHD intervention, metabolites of gut microbiota are enriched and increased in tryptophan metabolism (Figure 3), which are closely associated with depression-like behavior and microbiota biomarkers (Figure 4). Specifically, the antidepressant effect of SHD is manifested in the downregulation of KYN pathway and upregulation of AhR pathway in tryptophan metabolism (Figure 5). Based on the above, these findings indicate that SHD could regulate microbiota-derived tryptophan metabolites, which may be the potential mechanism for alleviating CUS-induced depressive-like behaviors.

Tryptophan metabolites have been proven to maintain intestinal epithelial barrier function by various mechanisms, such as regulating intestinal mucosa permeability and ameliorating intestinal barrier damage (Li et al., 2021; Wang et al., 2023). Among tryptophan metabolites, indoles play a crucial role in regulating intestinal integrity and modulating barrier function by binding to the AhR (Sun et al., 2020). TJs consist of peripheral membrane proteins (e.g., ZO-1), transmembrane proteins (e.g., Occludin and Claudin) and regulatory proteins (Kaminsky et al., 2021), which are the key determinant of intestinal barrier function (Tu et al., 2024). Occludin is considered as a critical regulator of the leak pathway (Zuo et al., 2020). ZO-1 is mainly involved in processes such as intestinal barrier signal transduction, cell proliferation and differentiation (Zuo et al., 2020). Intestinal permeability is a sign of an impaired intestinal barrier function (Schoultz and Keita, 2020). The results indicated that SHD could enhance intestinal mucosal barrier by downregulating the levels of LPS, D-LA and DAO in serum, upregulating the levels of ZO-1, Occludin in the colon (Figures 6A–G). The results suggest the beneficial effect of SHD on intestinal permeability and mucosal barrier.

Autophagy is crucial for maintaining intestinal mucosal barrier by regulating TJs (Wu et al., 2019). Autophagy is initiated by the serine/threonine kinase unc-51 like autophagy activating kinase 1(ULK1) complex (Umeda et al., 2006). The central upstream regulators of autophagy are mTOR and AMPK (Haq et al., 2019). mTOR is a serine/threonine kinase that regulates autophagic process negatively, whereas AMPK can trigger autophagy by the phosphorylation of ULK1 or through mTOR negative regulation (Xu and Wan, 2023). When autophagy is inhibited, as autophagy markers, the expressions of LC3, ATG5 and Beclin1 are reduced. However, the expression of p62 is increased (Changotra et al., 2022; Kumar et al., 2022; Prerna and Dubey, 2022; Yuan et al., 2022). A study has confirmed that Baitouweng Decoction could elevate the levels of ZO-1, Occludin, which are beneficial to repairing colonic barrier in colitis mice by means of regulating AMPK/mTOR-mediated autophagy (Pan et al., 2023).

Microbiota-derived metabolites from tryptophan exhibited intestinal protective effects resulting from maintaining gut mucosal barrier integrity and regulating the AMPK autophagy pathway (Gao et al., 2022), whereas the autophagy-deficient intestinal mucosal is more susceptible to injury (Pott et al., 2018). In addition, tryptophan metabolites, including tryptamine, IAA, IPA, indole, have an intimate link with the regulation of autophagy (Mizushima, 2009). Tryptophan accelerates the production of indole compounds, for instance, IAA, ILA and IAId, and activates AhR functional activity (Sun et al., 2020). KYN, the first major stable tryptophan metabolite, inhibits autophagy through the AhR (Mizushima, 2009). Furthermore tryptophan can play a role in alleviation of diarrhea by repairing intestine barrier involved mTOR pathway (Li et al., 2024b). After SHD treatment, the expressions of AMPK, LC3, ATG5 and Beclin1 were upregulated, and the expressions of mTOR, p62 were deregulated (Figures 6H–J). Figure 7 demonstrated that tryptophan metabolites in colonic contents were closely related to the intestinal barrier function and AMPK/mTOR pathway of autophagy. Our results further demonstrate that SHD might regulate the microbiota-derived tryptophan metabolism to trigger the AMPK/mTOR pathway of autophagy, enhancing the intestinal barrier, which can exert a therapeutic effect on depression-like behaviors.

**FIGURE 8:**
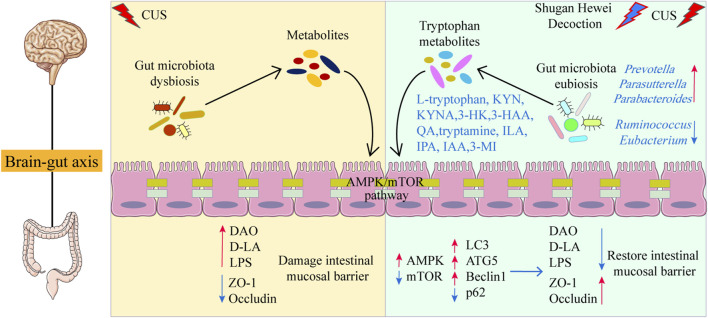
Schematic diagram of the mechanism of SHD in ameliorating depression-like behaviors.

In the research, we first verified the critical role and regulatory function of SHD by microbiota-derived tryptophan metabolism and AMPK/mTOR pathway of autophagy in improving depression-like behaviors and revealed its associated molecular mechanism (Figure 8). The effect of SHD was highly dependent on the tryptophan metabolism, AMPK/mTOR pathway of autophagy and intestinal barrier function. However, this study has some limitations. A series of in-depth studies need to be conducted, such as the interaction mechanism between gut microbiota, tryptophan metabolism and AMPK/mTOR pathway of autophagy. In the following experiments, first, the activity of individual components of SHD would be further verified on different parts of the central and peripheral nervous system, using spatial metabolomics, which would reveal the brain-gut-microbiota axis or microbe-metabolite axis communication mechanism of SHD. Second, we will verify the antidepressant effect of SHD depending on microbiota-derived metabolites using fecal microbiota transplantation and germ-free rats. Additionally, we would further reveal the gene structure and gene expression status of individual cells in the pathological mechanism of depression by single cell sequencing.

## 5 Conclusion

In conclusion, SHD could regulate microbiota-derived tryptophan metabolism, and trigger the AMPK/mTOR pathway of autophagy, enhancing the intestinal barrier function to improve depression-like behaviors in the CUS model. Our endeavor also provides novel insight into the antidepressant effect of this Chinese medicinal formula, which further corroborates the traditional application of SHD and contributes to its antidepressant pharmacological validation. These findings provide complementary and alternative treatments for strategy of depression.

## Data Availability

The datasets presented in this study can be found in the (online) repository, accession number (NCBI SRP466650). https://www.ncbi.nlm.nih.gov/search/all/?term=SRP466650.

## Abbreviations

3-HAA, 3-hyrdoxyanthranilic acid; 3-HK, 3-hydroxy-kynurenine; 3-MI, 3-methylindole; AMPK, adenosine monophosphate-activated protein kinase; ATG5, autophagy related protein 5; CUS, chronic unpredictable stress; DAO, diamine oxidase; D-LA, d-lactate; FOS, fructooligosaccharide; FST, forced swimming test; IAA, indole-3-acetic acid; IH, immunohistochemistry; ILA, indole-3-lactic acid; IPA, indole-3-propionic acid; KYN, kynurenine; KYNA, kynurenic acid; LC3, microtubule associated protein light chain 3; LC-MS/MS, liquid chromatography-mass/mass spectrometry; LPS, lipopolysaccharide; mTOR, mammalian target of rapamycin; OPT, open-field test; QA, quinolinic acid; SHD, Shugan Hewei Decoction; SPT, sucrose preference test; TJs, Tight junctions; ZO-1, zonula occludens 1.

## References

[B1] Agus A. Planchais J. Sokol H. (2018). Gut microbiota regulation of tryptophan metabolism in health and disease. Cell Host Microbe 23 (6), 716–724. 10.1016/j.chom.2018.05.003 29902437

[B2] Berwian I. M. Walter H. Seifritz E. Huys Q. J. (2017). Predicting relapse after antidepressant withdrawal - a systematic review. Psychol. Med. 47 (3), 426–437. 10.1017/S0033291716002580 27786144 PMC5244448

[B3] Changotra H. Kaur S. Yadav S. S. Gupta G. L. Parkash J. Duseja A. (2022). ATG5: a central autophagy regulator implicated in various human diseases. Cell Biochem. Funct. 40 (7), 650–667. 10.1002/cbf.3740 36062813

[B4] Chen L. M. Bao C. H. Wu Y. Liang S. H. Wang D. Wu H. G. (2021). Tryptophan-kynurenine metabolism: a link between the gut and brain for depression in inflammatory bowel disease. J. Neuroinflammation 18 (1), 135. 10.1186/s12974-021-02175-2 34127024 PMC8204445

[B5] Chi L. Khan I. Lin Z. Zhang J. Lee M. Zheng Y. (2020). Fructo-oligosaccharides from Morinda officinalis remodeled gut microbiota and alleviated depression features in a stress rat model. Phytomedicine 67, 153157. 10.1016/j.phymed.2019.153157 31896054

[B6] Cruz-Pereira J. S. Rea K. Nolan Y. M. O'Leary O. F. Dinan T. G. Cryan J. F. (2020). Depression's unholy trinity: dysregulated stress, immunity, and the microbiome. Annu. Rev. Psychol. 71, 49–78. 10.1146/annurev-psych-122216-011613 31567042

[B7] Deng Y. Zhou M. Wang J. Yao J. Yu J. Gao R. (2021). Involvement of the microbiota-gut-brain axis in chronic restraint stress: disturbances of the kynurenine metabolic pathway in both the gut and brain. Gut Microbes 13 (1), 1–16. 10.1080/19490976.2020.1869501 PMC7872056 33535879

[B8] Fan Q. Guan X. Hou Y. Liu Y. Wei W. Hao H. (2020). Paeoniflorin modulates gut microbial production of indole-3-lactate and epithelial autophagy to alleviate colitis in mice. Phytomedicine 79, 153345. 10.1016/j.phymed.2020.153345 33002829

[B9] Fernandez-Cantos M. V. Babu A. F. Hanhineva K. Kuipers O. P. (2024). Identification of metabolites produced by six gut commensal Bacteroidales strains using non-targeted LC-MS/MS metabolite profiling. Microbiol. Res. 283, 127700. 10.1016/j.micres.2024.127700 38518452

[B10] Foerster E. G. Mukherjee T. Cabral-Fernandes L. Rocha J. Girardin S. E. Philpott D. J. (2022). How autophagy controls the intestinal epithelial barrier. Autophagy 18 (1), 86–103. 10.1080/15548627.2021.1909406 33906557 PMC8865220

[B11] Gao K. Mu C. L. Farzi A. Zhu W. Y. (2020). Tryptophan metabolism: a link between the gut microbiota and brain. Adv. Nutr. 11 (3), 709–723. 10.1093/advances/nmz127 31825083 PMC7231603

[B12] Gao N. Yang Y. Liu S. Fang C. Dou X. Shan A. (2022). Gut-derived metabolites from dietary tryptophan supplementation quench intestinal inflammation through the AMPK-SIRT1-autophagy pathway. J. Agric. Food Chem. 70 (51), 16080–16095. 10.1021/acs.jafc.2c05381 36521060

[B13] Gasaly N. de Vos P. Hermoso M. A. (2021). Impact of bacterial metabolites on gut barrier function and host immunity: a focus on bacterial metabolism and its relevance for intestinal inflammation. Front. Immunol. 12, 658354. 10.3389/fimmu.2021.658354 34122415 PMC8187770

[B14] Gassen N. C. Rein T. (2019). Is there a role of autophagy in depression and antidepressant action? Front. Psychiatry 10, 337. 10.3389/fpsyt.2019.00337 31156481 PMC6529564

[B15] Ghosh S. Whitley C. S. Haribabu B. Jala V. R. (2021). Regulation of intestinal barrier function by microbial metabolites. Cell Mol. Gastroenterol. Hepatol. 11 (5), 1463–1482. 10.1016/j.jcmgh.2021.02.007 33610769 PMC8025057

[B16] Guo H. Ouyang Y. Yin H. Cui H. Deng H. Ouyang P. (2022). Induction of autophagy via the ROS-dependent AMPK-mTOR pathway protects copper-induced spermatogenesis disorder. Redox Biol. 49, 102227. 10.1016/j.redox.2021.102227 34979450 PMC8728583

[B17] Haq S. Grondin J. Banskota S. Khan W. I. (2019). Autophagy: roles in intestinal mucosal homeostasis and inflammation. J. Biomed. Sci. 26 (1), 19. 10.1186/s12929-019-0512-2 30764829 PMC6375151

[B67] James K. R. Gomes T. Elmentaite R. Kumar N. Gulliver E. L. Teichmann S. A. (2020). Distinct microbial and immune niches of the human colon. Nat Immunol. 21 (3), 343–353. 10.1038/s41590-020-0602-z 32066951 PMC7212050

[B18] Ju T. Kong J. Y. Stothard P. Willing B. P. (2019). Defining the role of Parasutterella, a previously uncharacterized member of the core gut microbiota. ISME J. 13 (6), 1520–1534. 10.1038/s41396-019-0364-5 30742017 PMC6776049

[B19] Kaminsky L. W. Al-Sadi R. Ma T. Y. (2021). IL-1β and the intestinal epithelial tight junction barrier. Front. Immunol. 12, 767456. 10.3389/fimmu.2021.767456 34759934 PMC8574155

[B20] Kumar A. V. Mills J. Lapierre L. R. (2022). Selective autophagy receptor p62/SQSTM1, a pivotal player in stress and aging. Front. Cell Dev. Biol. 10, 793328. 10.3389/fcell.2022.793328 35237597 PMC8883344

[B21] Li B. Yan Y. Zhang T. Xu H. Wu X. Wu L. L. (2024a). Quercetin reshapes gut microbiota homeostasis and modulates brain metabolic profile to regulate depression-like behaviors induced by CUMS in rats. Front. Pharmacol. 15, 1362464. 10.3389/fphar.2024.1362464 38595919 PMC11002179

[B22] Li J. Yan Y. Fu Y. Chen Z. Yang Y. Wang W. (2024b). ACE2 mediates tryptophan alleviation on diarrhea by repairing intestine barrier involved mTOR pathway. Cell Mol. Biol. Lett. 29 (1), 90. 10.1186/s11658-024-00603-8 38877403 PMC11179371

[B23] Li J. Zhang L. Wu T. Li Y. Zhou X. Ruan Z. (2021). Indole-3-propionic acid improved the intestinal barrier by enhancing epithelial barrier and mucus barrier. J. Agric. Food Chem. 69 (5), 1487–1495. 10.1021/acs.jafc.0c05205 33356219

[B24] Li Q. Hu J. J. Qiu Z. P. Li J. Liu S. L. Chen X. (2023). Shuganheweitang ameliorates chronic unpredictable mild stress-induced depression-like behaviors in rats through the PI3K/AKT/mTOR pathway: involvement of amino Acids, glycerophospholipids, and energy metabolism. Chin. Med. 14, 13–55. 10.4236/cm.2023.141002

[B25] Li Y. Liu N. Ge Y. Yang Y. Ren F. Wu Z. (2022). Tryptophan and the innate intestinal immunity: crosstalk between metabolites, host innate immune cells, and microbiota. Eur. J. Immunol. 52 (6), 856–868. 10.1002/eji.202149401 35362153

[B26] Ling C. Versloot C. J. Arvidsson K. M. Hu G. Swain N. Bandsma R. (2023). Rebalancing of mitochondrial homeostasis through an NAD (+)-SIRT1 pathway preserves intestinal barrier function in severe malnutrition. EBioMedicine 96, 104809. 10.1016/j.ebiom.2023.104809 37738832 PMC10520344

[B27] Liu D. Zhang S. Li S. Zhang Q. Cai Y. Xie Z. (2023a). Indoleacrylic acid produced by Parabacteroides distasonis alleviates type 2 diabetes via activation of AhR to repair intestinal barrier. BMC Biol. 21 (1), 90. 10.1186/s12915-023-01578-2 37072819 PMC10114473

[B28] Liu L. Wang H. Chen X. Zhang Y. Zhang H. Xie P. (2023b). Gut microbiota and its metabolites in depression: from pathogenesis to treatment. EBioMedicine 90, 104527. 10.1016/j.ebiom.2023.104527 36963238 PMC10051028

[B29] Lukic I. Getselter D. Ziv O. Oron O. Reuveni E. Elliott E. (2019). Antidepressants affect gut microbiota and Ruminococcus flavefaciens is able to abolish their effects on depressive-like behavior. Transl. Psychiatry 9 (1), 133. 10.1038/s41398-019-0466-x 30967529 PMC6456569

[B30] Luo D. Huang Z. Jia G. Zhao H. Liu G. Chen X. (2023). Naringin mitigates LPS-induced intestinal barrier injury in mice. Food Funct. 14 (3), 1617–1626. 10.1039/d2fo03586c 36688440

[B31] Machado-Vieira R. Zanetti M. V. Teixeira A. L. Uno M. Valiengo L. L. Marie S. K. (2015). Decreased AKT1/mTOR pathway mRNA expression in short-term bipolar disorder. Eur. Neuropsychopharmacol. 25 (4), 468–473. 10.1016/j.euroneuro.2015.02.002 25726893 PMC5863235

[B32] Malhi G. S. Mann J. J. (2018). Depression. Lancet 392 (10161), 2299–2312. 10.1016/S0140-6736(18)31948-2 30396512

[B33] Marwaha S. Palmer E. Suppes T. Cons E. Young A. H. Upthegrove R. (2023). Novel and emerging treatments for major depression. Lancet 401 (10371), 141–153. 10.1016/S0140-6736(22)02080-3 36535295

[B34] McCarron R. M. Shapiro B. Rawles J. Luo J. (2021). Depression. Ann. Intern Med. 174 (5), ITC65–ITC80. 10.7326/AITC202105180 33971098

[B35] McGuinness A. J. Davis J. A. Dawson S. L. Loughman A. Collier F. Jacka F. N. (2022). A systematic review of gut microbiota composition in observational studies of major depressive disorder, bipolar disorder and schizophrenia. Mol. Psychiatry 27 (4), 1920–1935. 10.1038/s41380-022-01456-3 35194166 PMC9126816

[B36] Mizushima N. (2009). Physiological functions of autophagy. Curr. Top. Microbiol. Immunol. 335, 71–84. 10.1007/978-3-642-00302-8_3 19802560

[B37] Mou X. J. Liu H. Lin N. Chen X. Liu S. L. (2019). Effects of Shugan Hewei Decoction and active substance fractions on behavior and neurotransmitter levels in hypothalamus of depression model rats. Zhongguo Zhong Yao Za Zhi 44 (15), 3343–3348. 10.19540/j.cnki.cjcmm.20190515.301 31602893

[B68] National Pharmacopoeia Commission (2020). Pharmacopoeia of the People’s Republic of China. China Medical Science and Technology Press, Beijing, 88, 108, 165, 258, 293.

[B38] Nikolova V. L. Smith M. Hall L. J. Cleare A. J. Stone J. M. Young A. H. (2021). Perturbations in gut microbiota composition in psychiatric disorders: a review and meta-analysis. JAMA Psychiatry 78 (12), 1343–1354. 10.1001/jamapsychiatry.2021.2573 34524405 PMC8444066

[B39] Pan S. M. Wang C. L. Hu Z. F. Zhang M. L. Pan Z. F. Chen B. (2023). Baitouweng decoction repairs the intestinal barrier in DSS-induced colitis mice via regulation of AMPK/mTOR-mediated autophagy. J. Ethnopharmacol. 318 (Pt A), 116888. 10.1016/j.jep.2023.116888 37437793

[B40] Pellegrini C. Fornai M. D'Antongiovanni V. Antonioli L. Bernardini N. Derkinderen P. (2023). The intestinal barrier in disorders of the central nervous system. Lancet Gastroenterol. Hepatol. 8 (1), 66–80. 10.1016/S2468-1253(22)00241-2 36334596

[B41] Pott J. Kabat A. M. Maloy K. J. (2018). Intestinal epithelial cell autophagy is required to protect against TNF-induced apoptosis during chronic colitis in mice. Cell Host Microbe 23 (2), 191–202. 10.1016/j.chom.2017.12.017 29358084

[B42] Prerna K. Dubey V. K. (2022). Beclin1-mediated interplay between autophagy and apoptosis: new understanding. Int. J. Biol. Macromol. 204, 258–273. 10.1016/j.ijbiomac.2022.02.005 35143849

[B43] Russell W. R. Duncan S. H. Scobbie L. Duncan G. Cantlay L. Flint H. J. (2013). Major phenylpropanoid-derived metabolites in the human gut can arise from microbial fermentation of protein. Mol. Nutr. Food Res. 57 (3), 523–535. 10.1002/mnfr.201200594 23349065

[B44] Schoultz I. Keita A. V. (2020). The intestinal barrier and current techniques for the assessment of gut permeability. Cells 9 (8), 1909. 10.3390/cells9081909 32824536 PMC7463717

[B45] Simpson C. A. Diaz-Arteche C. Eliby D. Schwartz O. S. Simmons J. G. Cowan C. (2021). The gut microbiota in anxiety and depression - a systematic review. Clin. Psychol. Rev. 83, 101943. 10.1016/j.cpr.2020.101943 33271426

[B46] Sun M. Ma N. He T. Johnston L. J. Ma X. (2020). Tryptophan (Trp) modulates gut homeostasis via aryl hydrocarbon receptor (AhR). Crit. Rev. Food Sci. Nutr. 60 (10), 1760–1768. 10.1080/10408398.2019.1598334 30924357

[B47] Taylor A. M. Thompson S. V. Edwards C. G. Musaad S. Khan N. A. Holscher H. D. (2020). Associations among diet, the gastrointestinal microbiota, and negative emotional states in adults. Nutr. Neurosci. 23 (12), 983–992. 10.1080/1028415X.2019.1582578 30794085

[B48] Trzeciak P. Herbet M. (2021). Role of the intestinal microbiome, intestinal barrier and psychobiotics in depression. Nutrients 13 (3), 927. 10.3390/nu13030927 33809367 PMC8000572

[B49] Tu J. Jiang Y. Tu L. Chen Y. Pan L. Feng D. (2024). Da-Cheng-Qi decoction improves severe acute pancreatitis capillary leakage syndrome by regulating tight junction-associated proteins. Front. Pharmacol. 15, 1138251. 10.3389/fphar.2024.1138251 38708079 PMC11066215

[B50] Umeda K. Ikenouchi J. Katahira-Tayama S. Furuse K. Sasaki H. Tsukita S. (2006). ZO-1 and ZO-2 independently determine where claudins are polymerized in tight-junction strand formation. Cell 126 (4), 741–754. 10.1016/j.cell.2006.06.043 16923393

[B51] Wan L. Qian C. Yang C. Peng S. Dong G. Jiang Z. (2024). Ginseng polysaccharides ameliorate ulcerative colitis via regulating gut microbiota and tryptophan metabolism. Int. J. Biol. Macromol. 265 (Pt 2), 130822. 10.1016/j.ijbiomac.2024.130822 38521337

[B52] Wang X. Huang S. Zhang M. Su Y. Pan Z. Luo X. (2023). Gegen Qinlian decoction activates AhR/IL-22 to repair intestinal barrier by modulating gut microbiota-related tryptophan metabolism in ulcerative colitis mice. J. Ethnopharmacol. 302 (Pt B), 115919. 10.1016/j.jep.2022.115919 36356716

[B53] Wang Y. S. Shen C. Y. Jiang J. G. (2019). Antidepressant active ingredients from herbs and nutraceuticals used in TCM: pharmacological mechanisms and prospects for drug discovery. Pharmacol. Res. 150, 104520. 10.1016/j.phrs.2019.104520 31706012

[B54] Willner P. Towell A. Sampson D. Sophokleous S. Muscat R. (1987). Reduction of sucrose preference by chronic unpredictable mild stress, and its restoration by a tricyclic antidepressant. Psychopharmacol. Berl. 93 (3), 358–364. 10.1007/BF00187257 3124165

[B55] Wu Y. Tang L. Wang B. Sun Q. Zhao P. Li W. (2019). The role of autophagy in maintaining intestinal mucosal barrier. J. Cell Physiol. 234 (11), 19406–19419. 10.1002/jcp.28722 31020664

[B56] Xie L. W. Cai S. Lu H. Y. Tang F. L. Zhu R. Q. Li M. (2024). Microbiota-derived I3A protects the intestine against radiation injury by activating AhR/IL-10/Wnt signaling and enhancing the abundance of probiotics. Gut Microbes 16 (1), 2347722. 10.1080/19490976.2024.2347722 38706205 PMC11086037

[B57] Xu Y. Wan W. (2023). Acetylation in the regulation of autophagy. Autophagy 19 (2), 379–387. 10.1080/15548627.2022.2062112 35435793 PMC9851266

[B58] Ye L. Wu J. Liu Z. Deng D. Bai S. Zhao J. (2023). Si-Ni-San alleviates early life stress-induced depression-like behaviors in adolescence via modulating Rac1 activity and associated spine plasticity in the nucleus accumbens. Front. Pharmacol. 14, 1274121. 10.3389/fphar.2023.1274121 38026979 PMC10646421

[B59] Yu J. B. Zhao Z. X. Peng R. Pan L. B. Fu J. Wang Y. (2019). Gut microbiota-based pharmacokinetics and the antidepressant mechanism of paeoniflorin. Front. Pharmacol. 10, 268. 10.3389/fphar.2019.00268 30949054 PMC6435784

[B60] Yuan J. Zhang Q. Chen S. Yan M. Yue L. (2022). LC3-associated phagocytosis in bacterial infection. Pathogens 11 (8), 863. 10.3390/pathogens11080863 36014984 PMC9415076

[B61] Yue Y. Chen Y. Liu H. Xu L. Zhou X. Liu S. L. (2021). Shugan Hewei Decoction alleviates cecum mucosal injury and improves depressive- and anxiety-like behaviors in chronic stress model rats by regulating cecal microbiota and inhibiting NLRP3 inflammasome. Front. Pharmacol. 12, 766474. 10.3389/fphar.2021.766474 34987395 PMC8721152

[B62] Zhang M. Li A. Yang Q. Li J. Zheng L. Liu L. (2023). Matrine alleviates depressive-like behaviors via modulating microbiota-gut-brain axis in CUMS-induced mice. J. Transl. Med. 21 (1), 145. 10.1186/s12967-023-03993-z 36829227 PMC9951532

[B63] Zhao X. Zeng H. Lei L. Tong X. Yang L. Zeng Q. (2021). Tight junctions and their regulation by non-coding RNAs. Int. J. Biol. Sci. 17 (3), 712–727. 10.7150/ijbs.45885 33767583 PMC7975691

[B64] Zheng P. Yang J. Li Y. Wu J. Liang W. Wang G. (2020). Gut microbial signatures can discriminate unipolar from bipolar depression. Adv. Sci. (Weinh) 7 (7), 1902862. 10.1002/advs.201902862 32274300 PMC7140990

[B65] Zhou J. Cai H. Duan Y. Pei K. Fan K. L. Liu J. (2018). Research progress on antidepressant effects of Sini San based on three progressive levels of “single herb, herb-pair, and complicated Chinese herbal formula”. Zhongguo Zhong Yao Za Zhi 43 (1), 46–51. 10.19540/j.cnki.cjcmm.20171106.014 29552810

[B66] Zuo L. Kuo W. T. Turner J. R. (2020). Tight junctions as targets and effectors of mucosal immune homeostasis. Cell Mol. Gastroenterol. Hepatol. 10 (2), 327–340. 10.1016/j.jcmgh.2020.04.001 32304780 PMC7326733

