# Supplementary material for: Microbiota-derived tryptophan metabolism and AMPK/mTOR pathway mediate antidepressant-like effect of Shugan Hewei Decoction
Source: Front Pharmacol. 2024 Sep 16;15:1466336. doi: 10.3389/fphar.2024.1466336 (PMC11439769; doi:10.3389/fphar.2024.1466336)
Supplement: Supplementary file 1 [file DataSheet1.docx]

Supplementary Material

# Supplementary Methods

## Preparation method of SHD

## Behavioral testing

## Shotgun metagenomic sequencing

## LC-MS/MS untargeted metabolomics

## Tryptophan metabolism by LC–MS/MS targeted metabolomics

# Supplementary Figures

## Figure S1. HPLC-Q-TOF-MS/MS analysis of ingredients from the SHD sample.

## Figure S2. Comparison of the relative microbiota abundance (phylum level) in the feces from the rats in each group

## Figure S3. OPLS-DA analysis and 200 permutation tests for fecal samples in positive and negative ion mode

## Figure S4. WB results of AMPK, p-AMPK, mTOR, p-mTOR, LC3, ATG5, Beclin1 and p62 at the colonic tissue of rats in each group

# Supplementary Tables

## Table S1 Compositional identification of SHD

## Table S2. Schedule of CUS procedures

## Table S3. Sequence list of primers used for RT-qPCR

## Table S4 Effect of SHD on the weight, SPT and FST of rats in each group

## Table S5. Effect of SHD on the OFT of rats in each group

## Table S6.

## Table S7.

## Table S8.Effect of SHD on LPS, D-LA and DAO at the serum of rats in each group

## Table S9. Effect of SHD on ZO-1 and Occludin at the colonic tissue of rats in each group

## Table S10. Effect of SHD on positive expression area of ZO-1 and Occludin at the colonic tissue of rats in each group (%)

## Table S11. Effect of SHD on AMPK, mTOR, LC3, ATG5, Beclin1 and p62 mRNA at the colonic tissue of rats in each group

## Table S12. Effect of SHD on the relative expression levels of p-AMPK/AMPK, p-mTOR/mTOR, LC3, ATG5, Beclin1 and p62 at the colonic tissue of rats in each group

# Supplementary Methods

## Preparation method of SHD

SHD raw herb weights in total of 930 g (10 doses of raw herbs of SHD) was soaked and then extracted with 10-fold mass of water (9,300 ml). First, 1.38 g of volatile oil (yield of 0.15%) and 23.7 g of aromatic water (yield of 2.55%) were extracted by steam distillation and then stored at −20°C; then by water extraction and alcohol precipitation method, the raw herb was boiled for 3 times, 2 h each, the water exact from each time were retrieved and mixed, then concentrated down to 930 ml (equal to 1 g crude herb/ml) by water bath method. The water extract was filtrated and 74.20 g of polysaccharides was obtained (yield of 7.98%), and the remaining liquid supernatant was concentrated by rotary evaporator and 242.20 g of extractum was obtained (yield of 26.0%); the polysaccharides and extractum were kept in a desiccator before use. The SHD total extract was obtained with a yield of 36.7% (341.48 g: 930 g). Distilled water was used to solve SHD total extract into liquid with the concentration of 1.12 g crude drug/ml (SHD-L) and 2.24 crude drug/ml (SHD-H). The FOS solution is prepared according to the relevant literature(Chi et al.,2020), with the concentration of 0.315 g/ml. After completion, all the solution was divided and sterilized, and then stored at 4℃ for later. The intragastric administration dosage was calculated based on the clinical equivalent dosage for an adult. The FOS was administered intragastrically (3.15 g/kg/day). The dosage of SHD-L and SHD-H were 2.68 g/kg/day (7.34 g crude drug/kg/d) and 5.36 g/kg/day (14.68 g crude drug/kg/d) and the dosage of SHD-L was the clinical equivalent. The control group rats were given free access to food and water, and five rats were housed in each cage.

## Behavioral testing

### Sucrose preference test

Sucrose preference test (SPT) has been designed to reflect the degree of anhedonia in the experimental subject. Rats were trained to adapt to sugar water the day before the experiment. Then these rats were deprived of water and food for 24 h. Subsequently these rats were given a free choice of two bottles of liquid (one with 1% sucrose solution, the other with water) for 2 h. During the test the position of the water bottle was exchanged for 1 h. Sucrose preference ratio (%) =sucrose intake (ml) × 100%/ [sucrose intake (ml) + water intake (ml)].

### Open-field test

Open-field test (OFT) was performed to the curiosity, exploration and locomotion of rats in a new environment, which was conducted followed SPT. The rat individually placed in the center of a 90 cm × 90 cm× 45 cm field reaction box was allowed to explore with freedom for 5 min during the OFT. The total movement distance, stationary time, and number of times that the rats crossed the central areas during the final 3 min were measured by a SMART 3.0 animal behavior video acquisition system (Panlab, Barcelona, Spain). The experimental area was sprayed withing 75% ethanol and wiped to avoid odor interference after each rat completed the test.

### Forced swimming test

Forced swimming test (FST) was conducted to detect the extent of despair of rat during a forced swim, as we described previously. Rat was allowed for a free swimming one day before the formal FST test for 15 min. During the experiment, the rats were separately forced to swim for 6 min in a clear cylinder filled with water at a temperature of 25 ± 1 °C. After 1 min of acclimatization, the total immobility time described as the amount of time that the rats spent keeping their heads above the water without struggling in the remaining 5 min was recorded and measured independently by two trained observers.

## Shotgun metagenomic sequencing

### Sequence quality control and genome assembly

The data were analyzed on the free online platform of Majorbio Cloud Platform (www.majorbio.com). Briefly, the paired-end Illumina reads were trimmed of adaptors, and low-quality reads (length<50 bp or with a quality value <20 or having N bases) were removed by fastp (https://github.com/OpenGene/fastp, version 0.20.0). Contigs with a length ≥ 300 bp were selected as the final assembling result, and then the contigs were used for further gene prediction and annotation.

### Gene prediction, taxonomy, and functional annotation

Open reading frames (ORFs) in contigs were identified using MetaGene (http://metagene.cb.k.u-tokyo.ac.jp/). The predicted ORFs with lengths being or over 100 bp were retrieved and translated into amino acid sequences. A non-redundant gene catalog was constructed using CD-HIT (http://www.bioinformatics.org/cd-hit/, version 4.6.1) with 90% sequence identity and 90% coverage. High-quality reads were aligned to the non-redundant gene catalogs to calculate gene abundance with 95% identity using SOAPaligner (http://soap.genomics.org.cn/, version 2.21). The taxonomic and KEGG annotations were conducted using Diamond (http://www.diamondsearch.org/index.php, version 0.8.35) against the Kyoto Encyclopedia of Genes and Genomes database (http://www.genome.jp/keeg/) with an e-value cutoff of 1e^-5^.

## LC-MS/MS untargeted metabolomics

### LC-MS/MS analysis

Chromatographic conditions：2μL of sample was separated by HSS T3 column (100 mm × 2.1 mm i.d., 1.8 μm) and then entered into mass spectrometry detection. The mobile phases consisted of 0.1% formic acid in water: acetonitrile (95:5, v/v) (solvent A) and 0.1% formic acid in acetonitrile: isopropanol: water (47.5:47.5:5, v/v) (solvent B). The solvent gradient changed according to the following conditions: from 0 to 3.5 min, 0% B to 24.5% B (0.4 mL/min); from 3.5 to 5 min, 24.5% B to 65% B (0.4 mL/min); from 5 to 5.5 min, 65% B to 100% B (0.4 mL/min); from 5.5to 7.4 min, 100% B to 100% B (0.4 mL/min to 0.6 mL/min); from 7.4 to 7.6 min, 100% B to 51.5% B (0.6 mL/min); from 7.6 to 7.8 min, 51.5% B to 0% B (0.6 mL/min to 0.5 mL/min); from 7.8 to 9 min, 0% B to 0% B (0.5 mL/min to 0.4 mL/min); from 9 to 10 min, 0% B to 0% B (0.4 mL/min) for equilibrating the systems. The sample injection volume was 2 µL and the flow rate was set to 0.4 mL/min. The column temperature was maintained at 40 ^o^C. During the period of analysis, all these samples were stored at 4 ^o^C.

MS conditions: The mass spectrometric data was collected using a Thermo UHPLC-Q Exactive HF-X Mass Spectrometer equipped with an electrospray ionization (ESI) source operating in either positive or negative ion mode. The optimal conditions were set as followed: heater temperature, 425 ^o^C; Capillary temperature, 325 ^o^C; sheath gas flow rate, 50 arb; Aux gas flow rate, 13 arb; ion-spray voltage floating (ISVF), -3500V in negative mode and 3500V in positive mode, respectively; Normalized collision energy, 20-40-60V rolling for MS/MS. Full MS resolution was 60000, and MS/MS resolution was 7500. Data acquisition was performed with the Data Dependent Acquisition (DDA) mode. The detection was carried out over a mass range of 70-1050 m/z.

### Data preprocessing and annotation

The raw data of LC/MS is preprocessed by Progenesis QI (Waters Corporation, Milford, USA) software, and a three-dimensional data matrix in CSV format is exported. The information in this three-dimensional matrix includes: sample information, metabolite name and mass spectral response intensity. At the same time, the metabolites were searched and identified based on the main databases including HMDB (http://www.hmdb.ca/), Metlin (https://metlin.scripps.edu/), and Majorbio Database. Standardized metabolomics data were fed to ropls (R package, Version 1.6.2) for principal component analysis (PCA) and orthogonal least partial squares discriminant analysis (OPLS-DA).

### Differential metabolites analysis

The selection of significantly different metabolites was determined based on the Variable Importance in the Projection (VIP) obtained by the OPLS-DA model and the p-value of student’s t test, and the metabolites with VIP>1, *p*<0.05 were significantly different metabolites. In addition, scipy (Python packages, Version 1.0.0) (https://docs.scipy.org/doc/scipy/) was used to conduct pathway enrichment analysis for the resulting significant differential metabolites and KEGG (http://www.kegg.jp) was used to identify related pathways of differential metabolites.

## Tryptophan metabolism by LC–MS/MS targeted metabolomics

Chromatographic conditions: Waters ACQUITY UPLC ® HSS T3 column (150 × 2.1 mm, 1.8 μm, Waters, Milford, USA). The column temperature: 40 ^o^C. The mobile phase: (A) 0.1% formic acid in water; (B) 0.1% formic acid in acetonitrile.

MS conditions: A SCIEX 6500 QTRAP (SCIEX, USA) was applied for mass spectrometry in positive and negative modes. Curtain Gas was 35 psi. Collision Gas was medium. IonSpray Voltage was 5500/-4500 V, and temperature was 550 ^o^C. Ion Source Gas 1 and Ion Source Gas 2 were 50 psi.

Data acquisition and processing: The automatic identification and integration of each ion fragment in the AB Sciex quantification software OS with default parameters and assisted with manual inspection. A linear regression standard curve was drawn using the ratio of the peak area to the internal standard and the concentration of the analyte as the abscissa. Sample concentration calculation: substitute the ratio of the peak area of the sample analyte to the internal standard into the linear equation to calculate the concentration result.

# Supplementary Figures


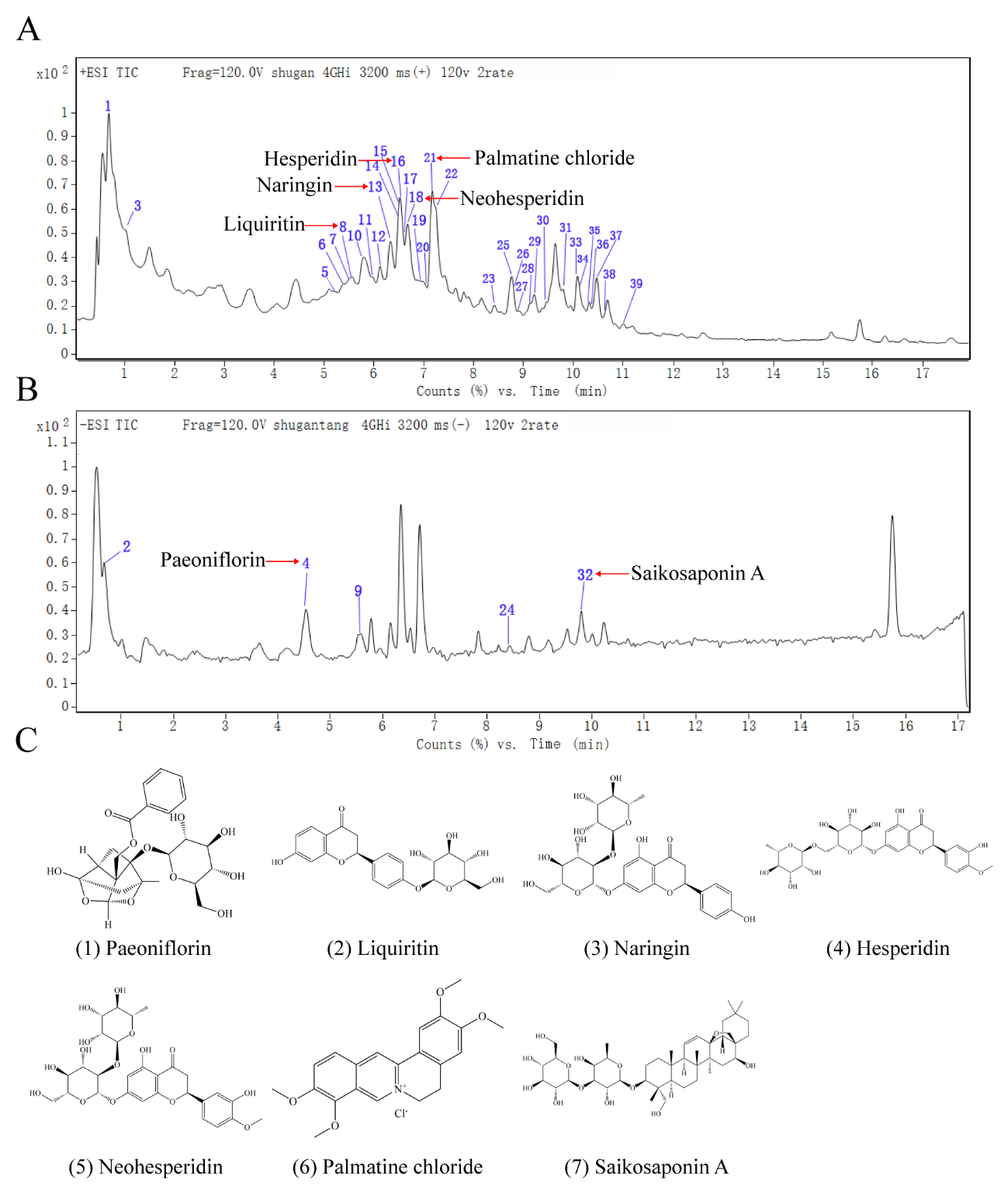


**Supplementary Figure S1.** HPLC-Q-TOF-MS/MS analysis of ingredients from the SHD sample. Total ion chromatograms of SHD in both positive (A) and negative modes (B). (C)the molecular structure of chemical composition.


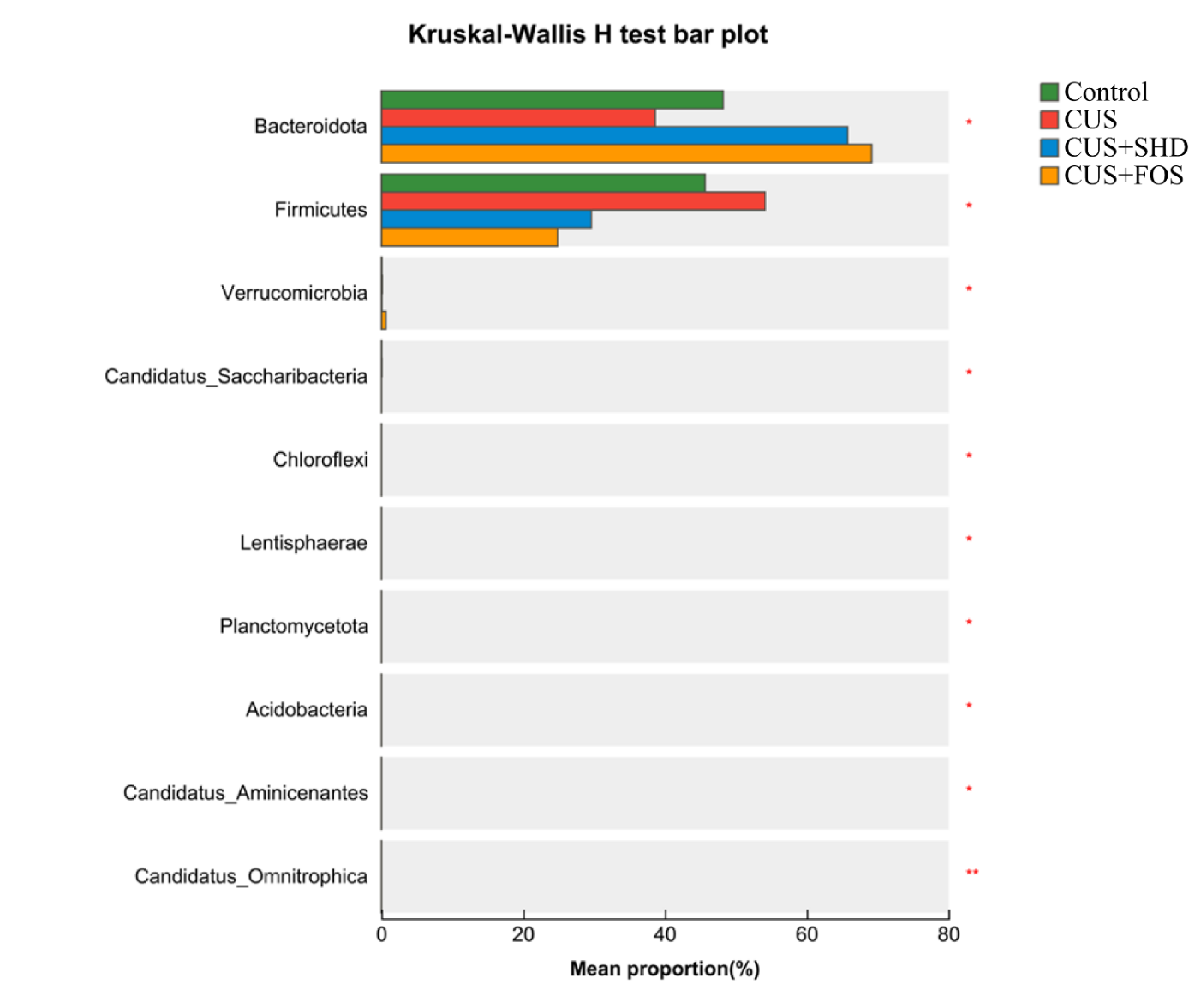
 **Figure S2.** Comparison of the relative microbiota abundance (phylum level) in the feces from the rats in each group (n=4)


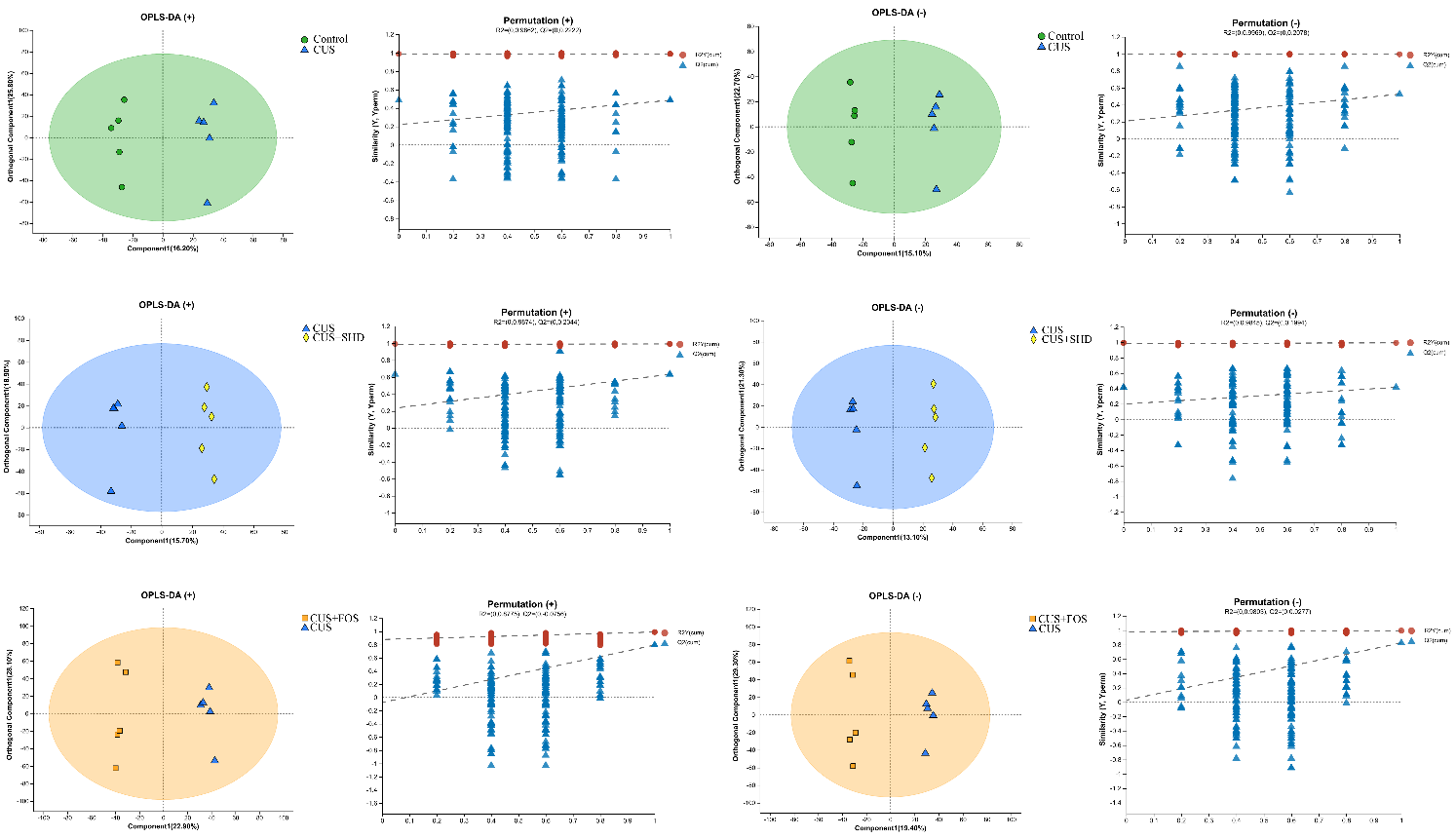


**Figure S3.** OPLS-DA analysis and 200 permutation tests for fecal samples in positive and negative ion mode (n=5)

| 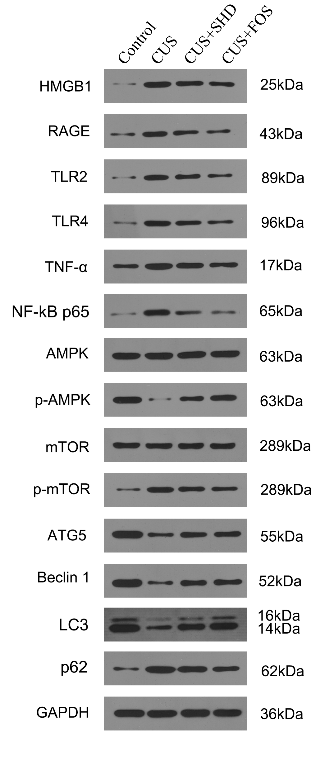  N=1 | 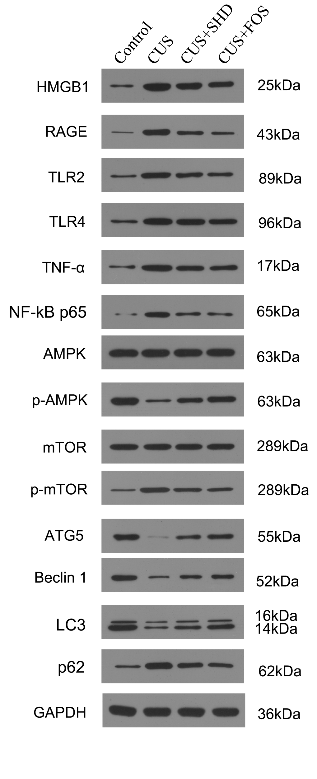  N=2 | 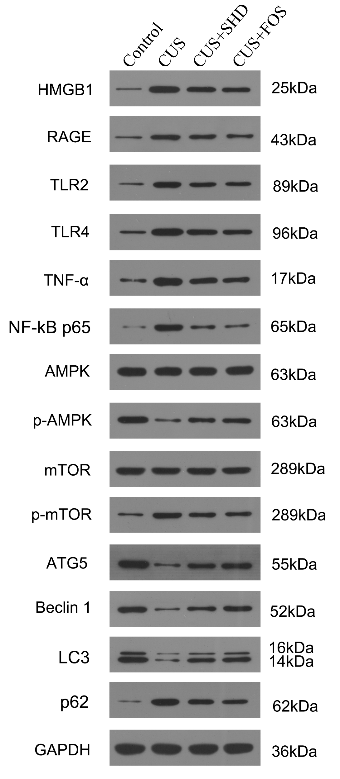  N=3 |
| --- | --- | --- |
| 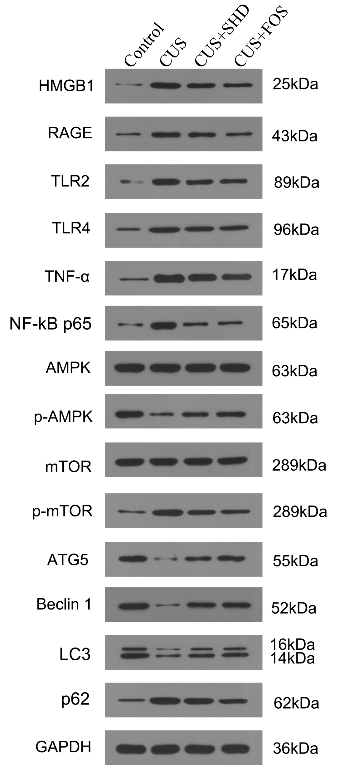  N=4 | 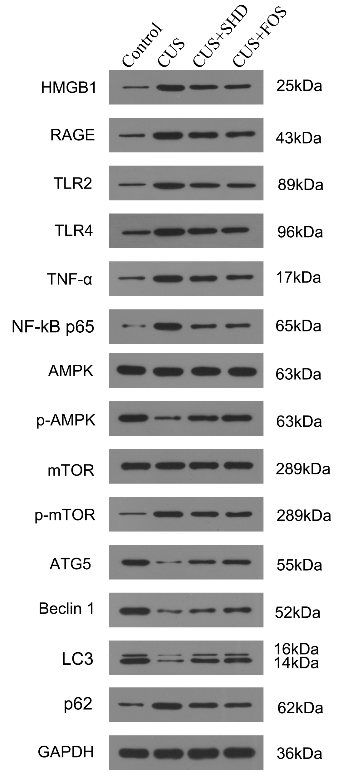  N=5 | 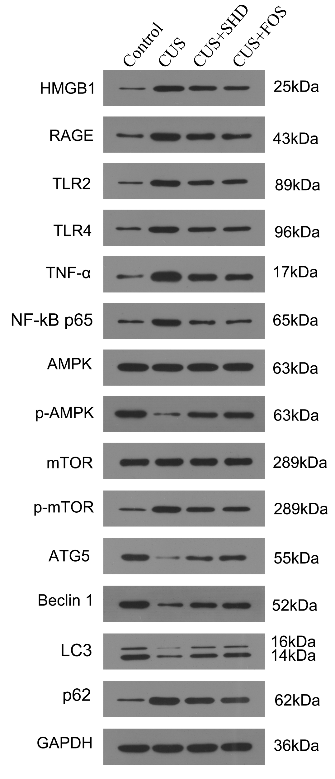  N=6 |
| 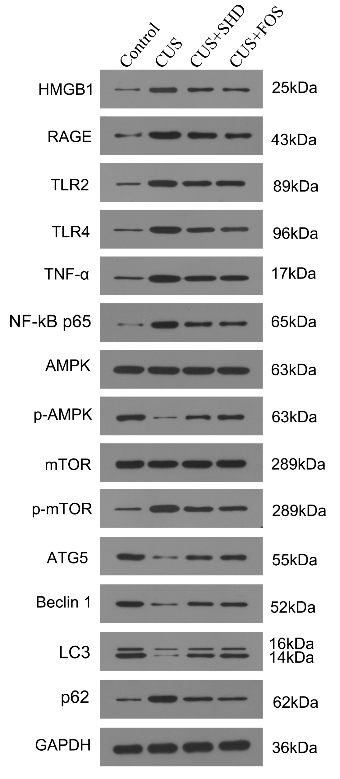  N=7 | 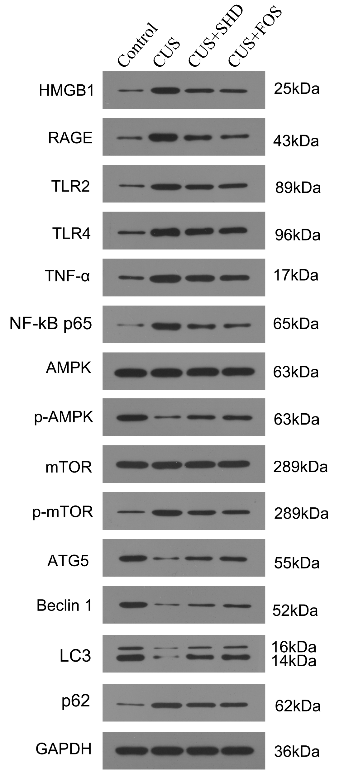  N=8 | 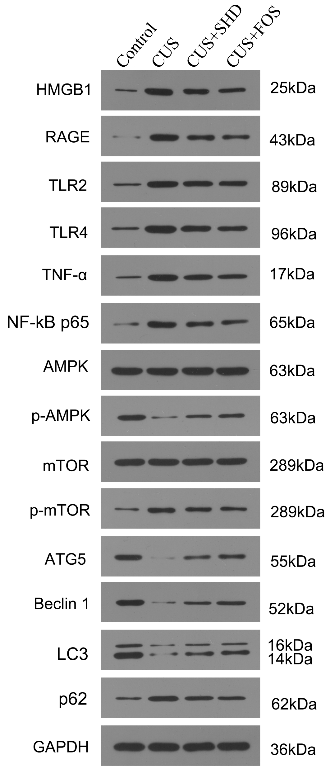  N=9 |

**Figure S4.** WB results of AMPK, p-AMPK, mTOR, p-mTOR, LC3, ATG5, Beclin1 and p62 at the colonic tissue of rats in each group (n=9)

# Supplementary Tables

**Table S1** **Compositional identification of SHD**

| Peak | Retention times/min | Identification | Molecular formula | Quasi-molecular ion（m/z） | Fragment  ion（m/z） | Error range  （10^-6^） | Ion  detection mode | Source |
| --- | --- | --- | --- | --- | --- | --- | --- | --- |
| 1 | 0.679 | N-(2-ethylaminobenzoyl)  tryptamine | C_18_H_19_N_3_O | 294.1642 | 144.1,116.08 | 13.94 | + | Euodiae Fructus |
| 2 | 0.824 | Gallic acid | C_7_H_6_O_5_ | 169.0135 | 125.02 | -4.14 | - | Paeoniae Radix Alba |
| 3 | 1.010 | Chlorogenic acid | C_16_H_18_O_9_ | 355.1021 | 163.04,145.03 | -0.84 | + | Euodiae Fructus |
| 4 | 4.464 | Paeoniflorin* | C_23_H_28_O_11_ | 525.1624 | 479.15,449.15 | 1.90 | - | Paeoniae Radix Alba |
| 5 | 5.224 | (R)-(+)-Canadine | C_20_H_21_NO_4_ | 340.1549 | 310.2 | 1.76 | + | Coptidis Rhizoma |
| 6 | 5.491 | Liquiritigenin | C_15_H_12_O_4_ | 257.0810 | 149.02,137.02 | 0.78 | + | Glycyrrhizae Radix et Rhizoma |
| 7 | 5.501 | Eriocitrin | C_27_H_32_O_15_ | 597.1781 | 289.07 | -5.53 | + | Glycyrrhizae Radix et Rhizoma |
| 8 | 5.530 | Liquiritin* | C_21_H_22_O_9_ | 419.1339 | 257.08,149.02 | 0.48 | + | Glycyrrhizae Radix et Rhizoma |
| 9 | 5.619 | Isoliquiritin apioside | C_26_H_30_O_13_ | 549.1607 | 417.12,255.07 | -1.27 | - | Glycyrrhizae Radix et Rhizoma |
| 10 | 5.807 | Berberrubine | C_19_H_16_ClNO_4_ | 322.1081 | 307.08,279.09 | 0.62 | + | Coptidis Rhizoma |
| 11 | 5.963 | Berlambine | C_20_H_17_NO_5_ | 352.1166 | 336.08,252.06 | -3.69 | + | Coptidis Rhizoma |
| 12 | 6.114 | Narirutin | C_27_H_32_O_14_ | 581.1878 | 435.13,419.13 | 2.24 | + | Aurantii Fructus Immaturus |
| 13 | 6.309 | Naringin* | C_27_H_32_O_14_ | 581.1854 | 419.13,273.07 | -1.89 | + | Aurantii Fructus Immaturus |
| 14 | 6.471 | Coptisine | C_19_H_14_NO_4_ | 321.0996 | 292.09,277.07 | 0.00 | + | Aurantii Fructus Immaturus |
| 15 | 6.508 | Dehydroevodiamine | C_19_H_15_N_3_O | 302.1295 | 286.09,258.10 | 2.32 | + | Euodiae Fructus |
| 16 | 6.513 | Hesperidin* | C_28_H_34_O_15_ | 611.1946 | 303.08,153.01 | -3.93 | + | Aurantii Fructus Immaturus |
| 17 | 6.559 | jatrorrhizine | C_20_H_20_INO_4_ | 338.1390 | 323.11,294.11 | -0.59 | + | Coptidis Rhizoma |
| 18 | 6.684 | Neohesperidin* | C_28_H_34_O_15_ | 611.1946 | 303.08,153.01 | -3.93 | + | Aurantii Fructus Immaturus |
| 19 | 6.965 | Worenine | C_20_H_16_NO_4_ | 334.1081 | 320.09,302.12 | 0.60 | + | Coptidis Rhizoma |
| 20 | 7.007 | evodianinine | C_19_H_13_N_3_O | 300.1136 | 285.09 | 1.67 | + | Euodiae Fructus |
| 21 | 7.139 | Palmatine chloride* | C_21_H_22_ClNO_4_ | 352.1566 | 337.13,322.11 | 4.83 | + | Coptidis Rhizoma |
| 22 | 7.238 | Berberine | C_20_H_18_NO_4_ | 336.1238 | 320.09,292.09 | 0.60 | + | Coptidis Rhizoma |
| 23 | 8.371 | Naringenin | C_15_H_12_O_5_ | 273.0757 | 153.13 | 0.00 | + | Aurantii Fructus Immaturus |
| 24 | 8.505 | Mudanpioside B | C_31_H_34_O_14_ | 629.1859 | 553.17,431.13 | -2.70 | - | Curcumae Radix |
| 25 | 8.740 | Costunolide | C_15_H_20_O_2_ | 233.1540 | 215.14,187.14 | 1.72 | + | Aucklandiae Radix |
| 26 | 8.785 | Procurcumadiol | C_15_H_22_O_3_ | 251.1631 | 197.13 | -4.38 | + | Curcumae Radix |
| 27 | 8.851 | Hesperetin | C_16_H_14_O_6_ | 303.0856 | 153.02 | -2.31 | + | Aurantii Fructus Immaturus |
| 28 | 9.174 | Licoricesaponin G2 | C_42_H_62_O_17_ | 839.4061 | 487.34,469.33 | 0.12 | + | Glycyrrhizae Radix et Rhizoma |
| 29 | 9.235 | Dihy-drocostunolide | C_15_H_22_O_2_ | 235.1683 | 188.96 | -4.25 | + | Aucklandiae Radix |
| 30 | 9.438 | Compound Glycyrrhizin | C_42_H_62_O_16_ | 823.4111 | 647.37 | 0.00 | + | Glycyrrhizae Radix et Rhizoma |
| 31 | 9.786 | Limonin | C_26_H_30_O_8_ | 471.2008 | 425.19,161.06 | -1.06 | + | Euodiae Fructus |
| 32 | 9.814 | Saikosaponin A* | C_42_H_68_O_13_ | 825.4623 | 779.5 | -2.30 | - | Bupleuri Radix |
| 33 | 10.045 | Nobiletin | C_21_H_22_O_8_ | 403.1395 | 373.09 | 1.98 | + | Aurantii Fructus Immaturus |
| 34 | 10.103 | Benzamide | C_19_H_21_N_3_O | 308.1738 | 165.73,134.06 | -6.17 | + | Euodiae Fructus |
| 35 | 10.314 | Evodiamine | C_19_H_17_N_3_O | 304.1429 | 171.09,134.06 | -4.93 | + | Euodiae Fructus |
| 36 | 10.350 | Atractylenolide I | C_15_H_18_O_2_ | 231.1371 | 217.10,163.07 | 27.69 | + | Atractylodis Macrocephalae Rhizoma |
| 37 | 10.403 | Tangeretin | C_20_H_20_O_7_ | 373.1265 | 343.08,135.04 | -4.56 | + | Aurantii Fructus Immaturus |
| 38 | 10.538 | Rutecarpine | C_18_H_13_N_3_O | 288.1127 | 273.08,169.07 | -1.39 | + | Euodiae Fructus |
| 39 | 10.986 | Atractylon | C_15_H_20_O | 217.1575 | 131.08,129.07 | -5.53 | + | Atractylodis Macrocephalae Rhizoma |

*indicated the components for reference substances.

**Table S2.** Schedule of CUS procedures

| Stressors | Day of CUS procedures | | | | | |
| --- | --- | --- | --- | --- | --- | --- |
| food deprivation 24 h | 10.30 | 11.7 | 11.10 | 11.17 | 11.25 | 12.3 |
| water deprivation 24 h | 11.1 | 11.4 | 11.13 | 11.16 | 11.29 | 12.5 |
| squeezing tail 1 min | 10.31 | 11.6 | 11.15 | 11.22 | 11.28 | 12.1 |
| swimming in 4℃water 5 min | 10.27 | 11.2 | 11.9 | 11.18 | 11.27 | 12.4 |
| shaking 1 time /s for 5 min | 10.26 | 11.5 | 11.14 | 11.21 | 11.26 | 12.2 |
| reversal of the light/dark | 10.28 | 11.8 | 11.11 | 11.20 | 11.24 | 12.6 |
| restraint for 2 h | 10.29 | 11.3 | 11.12 | 11.19 | 11.23 | 11.30 |

**Table S3.** Sequence list of primers used for qPCR

| Gene | Forward(5ʹ-3ʹ) | Reverse(3ʹ-5ʹ) | Length(bp) |
| --- | --- | --- | --- |
| AMPK | TACCTCGCCTCCAGTCC | GTGCTTTGGGGCTGTCT | 121 |
| mTOR | ACCAGGGGCTTCAACAG | TAACAGGATGGTGGAGTGC | 53 |
| LC3 | GCCGCCCTAAAGGTTACTGT | CTCGAGGTCCAACCCACAAA | 81 |
| ATG5 | CACTGGGACTTCTGCTCCTG | TCAACCAAGCCAAACCGAGG | 124 |
| Beclin1 | TTGCGTCAGCTCTCGTCAAG | GCCTTAGACCCCTCCATTCTTTA | 221 |
| p62 | GGAAGCTGAAACATGGGCAC | CAACCTCGATGCCCGACTC | 129 |
| GAPDH | GCATCTTCTTGTGCAGTGCC | ACCAGCTTCCCATTCTCAGC | 237 |

**Table S4.** Effect of SHD on the weight, SPT and FST of rats in each group (‾x ± SEM, n=10)

| Group | Weight(g) | Sucrose preference (%) | Immobility time (s) |
| --- | --- | --- | --- |
| Control | 419.50±6.05 | 77.14±3.38 | 51.00±6.55 |
| CUS | 335.40±5.67** | 42.53±4.46** | 151.80±12.25** |
| CUS+SHD-L | 391.80±6.00^##^ | 71.14±6.30^##^ | 60.80±5.763^##^ |
| CUS+SHD-H | 374.80±5.87^##^ | 55.00±6.63 | 110.8±10.39^#^ |
| CUS+FOS | 375.20±7.65^##^ | 65.87±4.49^##^ | 91.80±14.11^##^ |

The data are expressed as the mean ± SEM. **p* < 0.05，***p* < 0.01 vs. the control group. ^#^*p* < 0.05，^##^*p* < 0.01 vs. the CUS group. **^Δ^***p* ＜0.05, **^ΔΔ^***p* ＜0.01 vs. the CUS+FOS group.

**Table S5.** Effect of SHD on the OFT of rats in each group (‾x ± SEM, n=8~10)


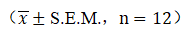

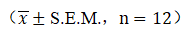


| Group | distance(cm) | zone crossing | duration(s) |
| --- | --- | --- | --- |
| Control | 490.40±54.02 | 21.38±1.05 | 98.03±12.10 |
| CUS | 317.00±44.73*** | 16.13±1.30*** | 190.30±7.23**** |
| CUS+SHD-L | 496.00±34.02^#^ | 20.88±0.90**^ΔΔ^** | 139.80±10.25^#^ |
| CUS+SHD-H | 385.10±44.47 | 18.25±0.75 | 133.50±10.14 |
| CUS+FOS | 465.80±32.64^#^ | 21.88±1.04^##^ | 145.30±8.04^#^ |

**Table S8.** Effect of SHD on LPS, D-LA and DAO at the serum of rats

in each group (‾x ± SEM, n=6)

| Group | LPS (ng/mL) | D-LA (μmol/L) | DAO (U/L) |
| --- | --- | --- | --- |
| Control | 1.03±0.05 | 4.75±0.13 | 30.52±1.29 |
| CUS | 4.96±0.09**** | 15.49±0.47**** | 126.79±6.17**** |
| CUS+SHD | 2.51±0.05^##ΔΔ^ | 7.95±0.21^##ΔΔ^ | 66.43±1.51^##ΔΔ^ |
| CUS+FOS | 3.10±0.06^##^ | 10.29±0.23^##^ | 83.33±2.04^##^ |

**Table S9.** Effect of SHD on ZO-1 and Occludin at the colonic tissue of rats in each group

(pg/mg prot) (‾x ± SEM, n=9)

| Group | ZO-1 | Occludin |
| --- | --- | --- |
| Control | 258.17±13.68 | 406.99±31.71 |
| CUS | 54.32±3.07**** | 76.74±3.64**** |
| CUS+SHD | 108.40±4.59^##ΔΔ^ | 142.91±5.23^##ΔΔ^ |
| CUS+FOS | 170.88±7.36^##^ | 212.34±8.83^##^ |

**Table S10.** Effect of SHD on positive expression area of ZO-1 and Occludin at the

colonic tissue of rats in each group (%) (‾x ± SEM, n=4)

| Group | ZO-1 | Occludin |
| --- | --- | --- |
| Control | 3.89±0.23 | 1.87±0.17 |
| CUS | 0.96±0.16**** | 0.36±0.16**** |
| CUS+SHD | 2.97±0.21^##^ | 1.56±0.17^##^ |
| CUS+FOS | 3.16±0.13^##^ | 1.87±0.22^##^ |

**Table S11.** Effect of SHD on AMPK, mTOR, LC3, ATG5, Beclin1 and p62 mRNA at the colonic tissue of rats in each group (‾x ± SEM, n=9)

| Group | AMPK | mTOR | LC3 | ATG5 | Beclin1 | P62 |
| --- | --- | --- | --- | --- | --- | --- |
| Control | 0.959±0.266 | 1.080±0.401 | 1.006±0.033 | 1.086±0.032 | 1.010±0.024 | 1.070±0.041 |
| CUS | 0.201±0.010**** | 4.219±0.076**** | 0.255±0.007**** | 0.247±0.012**** | 0.240±0.008**** | 4.431±0.094**** |
| CUS+SHD | 0.382±0.015^##ΔΔ^ | 3.516±0.086^##ΔΔ^ | 0.468±0.013^##ΔΔ^ | 0.457±0.018^##ΔΔ^ | 0.450±0.014^##ΔΔ^ | 3.674±0.110^##ΔΔ^ |
| CUS+FOS | 0.511±0.014^##^ | 2.167±0.033^##^ | 0.572±0.011^##^ | 0.591±0.014^##^ | 0.566±0.009^##^ | 2.039±0.052^##^ |

**Table S12.** Effect of SHD on the relative expression levels of p-AMPK/AMPK, p-mTOR/mTOR, LC3, ATG5, Beclin1 and p62 at the colonic tissue of rats in each group (‾x ± SEM, n=9)

| Group | p-AMPK/AMPK | p-mTOR/mTOR | LC3 | ATG5 | Beclin1 | P62 |
| --- | --- | --- | --- | --- | --- | --- |
| Control | 1.005±0.020 | 0.371±0.009 | 0.979±0.020 | 0.964±0.019 | 0.987±0.020 | 0.329±0.033 |
| CUS | 0.293±0.029**** | 1.053±0.028**** | 0.295±0.029**** | 0.231±0.038**** | 0.283±0.027**** | 0.999±0.014**** |
| CUS+SHD | 0.663±0.031^##Δ^ | 0.829±0.038^##^ | 0.664±0.031^##Δ^ | 0.623±0.018^##ΔΔ^ | 0.610±0.045^##^ | 0.766±0.037^##Δ^ |
| CUS+FOS | 0.786±0.033^##^ | 0.736±0.029^##^ | 0.778±0.038^##^ | 0.761±0.019^##^ | 0.723±0.041^##^ | 0.631±0.029^##^ |

**References**

Chi, L. D., Khan, I., Lin, Z. B., Zhang, J. W., Lee, M., Leong, W., Zheng, Y., et al. (2020). Fructo-oligosaccharides from Morinda officinalis remodeled gut microbiota and alleviated depression features in a stress rat model. *Phytomedicine*, 67, 153157. doi: 10.1016/j.phymed.2019.153157
